# The soybean *Rhg1* amino acid transporter gene alters glutamate homeostasis and jasmonic acid‐induced resistance to soybean cyst nematode

**DOI:** 10.1111/mpp.12753

**Published:** 2018-11-15

**Authors:** Wei Guo, Feng Zhang, Aili Bao, Qingbo You, Zeyu Li, Jingsheng Chen, Yihui Cheng, Wei Zhao, Xinjie Shen, Xinan Zhou, Yongqing Jiao

**Affiliations:** ^1^ Key Laboratory of Oil Crop Biology of the Ministry of Agriculture Oil Crops Research Institute of the Chinese Academy of Agricultural Sciences Wuhan Hubei 430062 China; ^2^ Daqing Branch of Heilongjiang Academy of Agricultural Sciences Daqing Heilongjiang 163316 China; ^3^ Collaborative Innovation Center of Henan Grain Crops, College of Agronomy Henan Agricultural University Zhengzhou Henan 450002 China

**Keywords:** amino acid transporter, glutamate, jasmonic acid, *Rhg1*, soybean, soybean cyst nematode

## Abstract

*Rhg1* (resistance to *Heterodera glycines* 1) is an important locus that contributes to resistance against soybean cyst nematode (SCN; *Heterodera glycines *Ichinohe), which is the most economically damaging disease of soybean worldwide. Simultaneous overexpression of three genes encoding a predicted amino acid transporter, an α‐soluble *N*‐ethylmaleimide‐sensitive factor attachment protein (α‐SNAP) and a predicted wound‐induced protein resulted in resistance to SCN provided by this locus. However, the roles of two of these genes (excluding α‐*SNAP*) remain unknown. Here, we report the functional characterization of *Glyma.18G022400*, a gene at the *Rhg1* locus that encodes the predicted amino acid transporter Rhg1‐GmAAT. Although the direct role of Rhg1‐GmAAT in glutamate transport was not demonstrated, multiple lines of evidence showed that Rhg1‐GmAAT impacts glutamic acid tolerance and glutamate transportation in soybean. Transcriptomic and metabolite profiling indicated that overexpression of *Rhg1‐GmAAT* activated the jasmonic acid (JA) pathway. Treatment with a JA biosynthesis inhibitor reduced the resistance provided by the *Rhg1*‐containing PI88788 to SCN, which suggested that the JA pathway might play a role in *Rhg1*‐mediated resistance to SCN. Our results could be helpful for the clarification of the mechanism of resistance to SCN provided by *Rhg1* in soybean.

## Introduction

Soybean cyst nematode (SCN; *Heterodera* *glycine *Ichinohe) is an important plant‐parasitic pest in soybean [*Glycine max* (L.) Merrill] and causes substantial damage to soybean production worldwide (Niblack *et al*., 2006). SCNs are obligate endoparasites that penetrate soybean roots and reprogram host root cells to form multinucleate cells, termed syncytia, which subsequently rob the infected plant of its nutrients for their own growth and reproduction (Gheysen and Mitchum, 2011). Other than rotation with non‐host crop plants, the breeding of SCN‐resistant soybean varieties is the most effective and environmentally friendly technique for the control of SCN (Cook, 2004).

The *Rhg1* (resistance to* Heterodera glycines* 1) locus, sometimes in combination with the *Rhg4* locus, provides the strongest known resistance to SCN (Brucker *et al*., 2005; Caldwell *et al*., 1960; Tylka *et al*., 2010; Webb *et al*., 1995) and has been widely used in the breeding of SCN‐resistant soybean cultivars. Two different alleles exist at the *Rhg1* locus: *Rhg1‐a*, which is from Peking, and *Rhg1‐b*, which is from PI88788 (Concibido *et al*., 2004; Kim *et al*., 2010). These two alleles have distinctive mechanisms of resistance to SCN. *Rhg1‐a* epistatically interacts with *Rhg4* in providing resistance to SCN race 3 (HG type 0), whereas *Rhg1‐b* functions independently (Brucker *et al*., 2005; Meksem *et al*., 2001). Three genes are responsible for the resistance provided by *Rhg1‐b*: *Glyma.18G022400 *(*Rhg1‐GmAAT*), which encodes a predicted amino acid transporter; *Glyma.18G022500 *(*GmSNAP18*), which encodes a predicted α‐soluble *N*‐ethylmaleimide‐sensitive factor (NSF) attachment protein (α‐SNAP); and *Glyma.18G022700 *(*Rhg1‐GmWI12*), which encodes a putative wound‐induced protein (Cook *et al*., 2012). The overexpression of any one of these three genes cannot provide resistance to SCN in soybean. However, the simultaneous overexpression of all three genes has been shown to account for resistance to SCN at *Rhg1* (Cook *et al*., 2012). Recently, the function of *GmSNAP18* by itself at *Rhg1‐b* has been investigated (Bayless *et al*., 2016; Liu *et al*., 2017); this gene encodes a dysfunctional α‐SNAP variant and results in the interruption of NSF function and vesicle trafficking in soybean. For the other two genes*, Rhg1*‐*GmAAT *and *Rhg1‐GmWI12*, resistance is attributable to elevated expression due to the increased copy number rather than nucleotide mutations. However, the characterization of these two genes, as well as the understanding of how all three genes coordinately function to provide resistance to SCN, is lacking.

Amino acids represent the main form of nitrogen transported by xylem and phloem among different organs in plants. In the roots, xylem loading involves membrane export from the stele cells into the xylem sap. In the shoots, apoplastic phloem loading from the leaf parenchyma involves export and import steps across membranes (Simpson, 1986). At the cellular level, amino acid transport across membranes is mediated by importers, exporters and facilitators (Okumoto and Pilot, 2011). Importers mediate transport towards the cytosol; exporters mediate transport away from the cytosol; in addition, importers and exporters mediate transport from or to the apoplasm, vacuoles or intracellular vesicles, respectively. More than 20 amino acid importers have been isolated and characterized, most of which are characterized as amino acid permeases (AAPs), cationic amino acid transporters (CATs) and lysine (Lys)/histidine (His) transporters (LHTs). All of these transporters mediate the proton‐dependent import of amino acids into the cell and have important biological functions in plants (Chen and Bush, 1997; Chen *et al*., 2001; Frommer *et al*., 1993, 1995; Hirner *et al*., 2006; Lee and Tegeder, 2004; Simpson, 1986). For example, *AtAAP1* and *AtLHT1* are responsible for the uptake of amino acids into root and mesophyll cells (Hirner *et al*., 2006; Lee *et al*., 2007; Svennerstam *et al*., 2008, 2011). *AtAAP2* and *AtAAP8* are involved in the accumulation of amino acids in developing seeds (Okumoto *et al*., 2002; Schmidt *et al*., 2007). In contrast with amino acid importers, few exporters have been identified (Okumoto and Pilot, 2011). The amino acid polyamine transporter (APC) *bidirectional amino acid transporter 1* (*BAT1*) may be an amino acid exporter. AtBAT1 exports glutamate and Lys and mediates the influx of γ‐aminobutyric acid (GABA), alanine (Ala) and arginine (Arg) (Dundar and Bush, 2009; Michaeli *et al*., 2011). A member of the drug and metabolite transporter superfamily, *siliques* *are red1* (*SiAR1*), functions as a bidirectional amino acid transporter and is responsible for amino acid homeostasis in developing siliques (Ladwig *et al*., 2012). The protein *u*sually *m*ultiple *a*cids *m*ove *i*n and out *t*ransporter 14 (UMAMIT14) is an amino acid exporter and is involved in the unloading of amino acids from the phloem in *Arabidopsis* roots (Besnard *et al*., 2016). In addition to their indispensable role as protein constituents, amino acids are critical metabolites that play prominent roles in many influential pathways in plants. For example, tryptophan (Trp) and methionine (Met) are precursors of auxins and ethylene (ET), respectively (Burstenbinder *et al*., 2007; Gfeller *et al*., 2010; Sauer *et al*., 2013). The *lysine histidine transporter 1* (*lht1*) mutant, which has a specific deficiency in its main physiological substrate, glutamine (Gln), shows stronger resistance to a broad spectrum of pathogens than does its wild‐type (WT) counterpart (Liu *et al*., 2010). In addition, treatment of *Arabidopsis* roots with Glu can activate blast resistance (Kadotani *et al*., 2016).

Jasmonic acid (JA), an important phytohormone involved in the regulation of plant development and defence, plays a crucial role in defence against plant‐parasitic nematodes (Avanci *et al*., 2010; Gfeller *et al*., 2010; Gleason *et al*., 2016; Matthews *et al*., 2014; Pauwels and Goossens, 2011). In *Arabidopsis*, the accumulation of the JA precursor *cis*‐(C)‐12‐oxo‐phytodienoic acid (OPDA), in addition to JA/jasmonoyl isoleucine (JA‐Ile), is beneficial for resistance against root‐knot nematodes (*Meloidogyne hapla*) (Gleason *et al*., 2016). In rice, treatment with methyl jasmonate (MeJA) can induce resistance to root‐knot nematodes (*Meloidogyne graminicola*) (Nahar *et al*., 2011). In soybean, overexpression of *Arabidopsis* JA biosynthesis genes provides modest resistance to SCN (Matthews *et al*., 2014). In contrast, the down‐regulation of both JA biosynthesis genes and JA signalling responses during SCN infection has been reported in susceptible soybean (Ithal *et al*., 2007a, 2007b). Together, these results indicate that the JA pathway is positively correlated with plant defence against nematodes.

In this study, we report the functional characterization of *Rhg1‐GmAAT*, one of three genes at the *Rhg1* locus responsible for resistance to SCN. Rhg1‐GmAAT is involved in Glu tolerance and glutamate transportation in soybean. Overexpression of *Rhg1‐GmAAT* can induce JA accumulation in transgenic soybean. Inhibition of JA biosynthesis by an inhibitor can reduce the resistance to SCN in the resistant *Rhg1*‐containing soybean germplasm PI88788, which indicates that JA might contribute to the resistance to SCN. Our study provides insights into the roles of *Rhg1‐GmAAT* in providing resistance to SCN and will be helpful in revealing the functional mechanism of *Rhg1*.

## Results

### *Rhg1‐GmAAT* is predicted to encode an amino acid transporter

Pfam on UniProt (the universal protein resource) predicted that the *Glyma.18G022400* gene, referred to as *Rhg1‐GmAAT*, at *Rhg1* encoded a 436‐amino‐acid protein that contained one amino acid transporter domain (from position 22 to 426). The alignment of protein sequences revealed that Rhg1‐GmAAT shared 58.72% amino acid identity with *Arabidopsis* vacuolar amino acid transporter 6C (AVT6C) and 58.31% amino acid identity with AtAVT6D. AtAVT6C and AtAVT6D are homologous to *Saccharomyces cerevisiae *AVT6 (Fig. S1, see Supporting Information), a vacuolar exporter of Glu and aspartic acid (Asp) (Chahomchuen *et al*., 2009).

**Figure 1:**
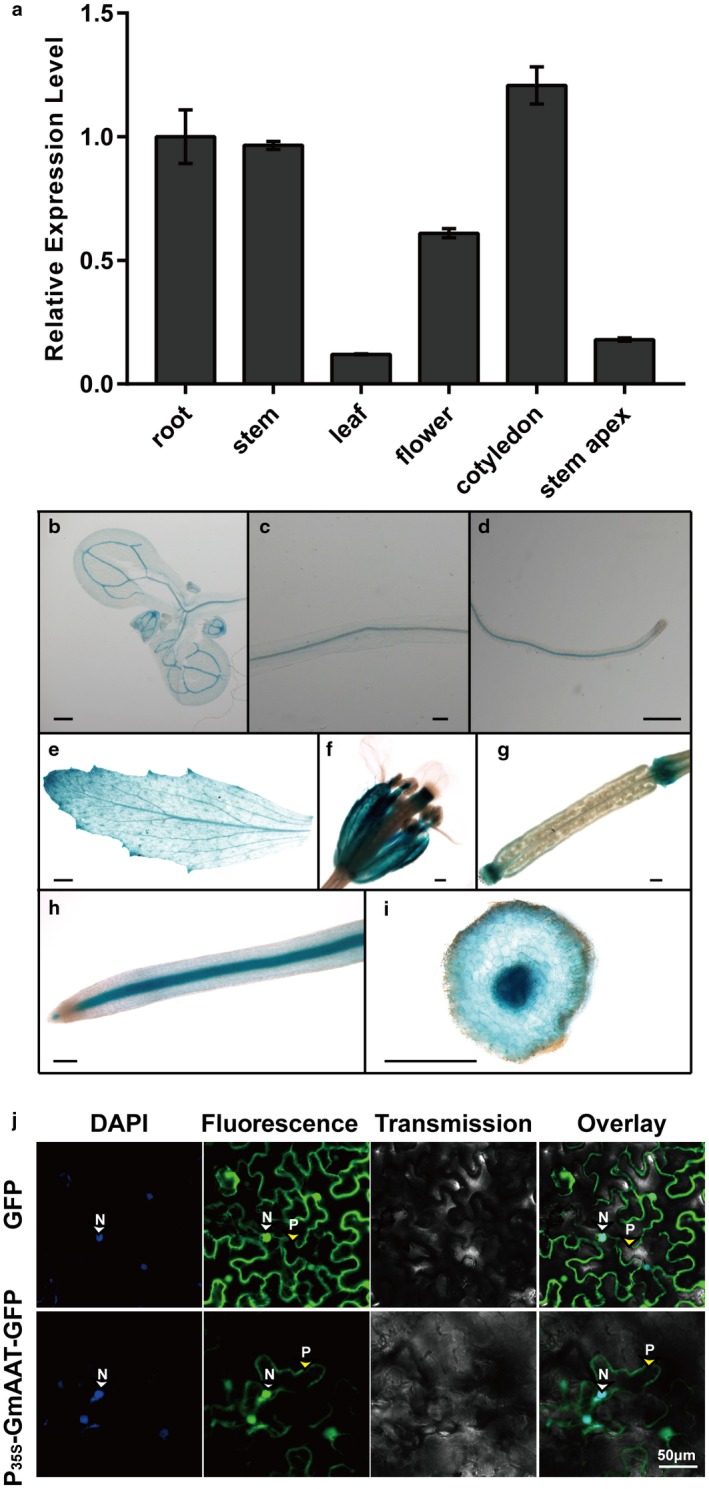
Expression and subcellular localization of Rhg1‐GmAAT. (a) The expression patterns of *Rhg1‐GmAAT* were determined by quantitative reverse transcription‐polymerase chain reaction (qRT‐PCR) and normalized to *SKIP16*. The values are the means ± standard deviations (SDs) (*n* = 3). (b–g) Histochemical analysis of β‐glucuronidase (GUS) activity from *Arabidopsis* plants expressing *P_GmAAT_‐GUS*. GUS staining in a 2‐week‐old cotyledon (b), 2‐week‐old stem (c), 2‐week‐old root (d), leaf of a 35‐day‐old plant (e), flower of a 35‐day‐old plant (scale bar, 10 mm) (f) and silique of a 35‐day‐old plant (g). (h, i) Histochemical analysis of GUS activity in soybean hairy roots expressing *P_GmAAT_‐GUS*. GUS staining in the stele (h) and within a root cross‐section (i). (j) Subcellular localization via a P_35S_‐GmAAT‐GFP fusion protein in tobacco epidermal leaf cells. From left to right: 4,6‐diamidino‐2‐phenylindole (DAPI)‐stained nuclear DNA, green fluorescent protein (GFP) fluorescence, bright‐field and overlay panels. Nucleus (N) and plasma membrane (P) are indicated by white and yellow arrows, respectively. Unless otherwise specified, scale bar = 200 μm. [Colour figure can be viewed at wileyonlinelibrary.com]

To determine the expression patterns of *Rhg1‐GmAAT* *in vivo*, we first used real‐time quantitative reverse transcription‐polymerase chain reaction (qRT‐PCR) to measure *Rhg1‐GmAAT* expression. *Rhg1‐GmAAT* was ubiquitously expressed in the roots, stems, leaves, flowers, cotyledons and stem apex, with the expression of *Rhg1‐GmAAT* being highest in the roots, stems and cotyledons (Fig. 1a). To investigate the expression pattern of *Rhg1‐GmAAT* in more detail, we fused the *Rhg1‐GmAAT* native promoter to a β‐glucuronidase (GUS) reporter and transformed the construct into *Arabidopsis*. Histochemical analyses of the transgenic *Arabidopsis* revealed that the promoter of the *Rhg1‐GmAAT* gene was active in the vasculature throughout the whole plant (i.e. roots, leaves, flowers, stems and siliques) (Fig. 1b–g). We also performed a histochemical analysis with the P_GmAAT_‐GUS construct in transgenic soybean hairy roots. GUS staining revealed a relatively strong signal in the stele of the transgenic hairy roots (Fig. 1h) and a highly enriched signal in the vascular tissues and pericycle (Fig. 1i). To verify this observation, more experiments are needed in the future. GUS expression supports the assumed role of Rhg1‐GmAAT in the transportation of amino acids between the organs of the plant via the vascular system. Amino acids are transported not only across the plasma membrane, but also in and out of all cellular compartments; as such, by overexpression of a P_35S_‐GmAAT‐green fluorescent protein (GFP) construct in tobacco epidermal leaf cells, onion epidermal cells and soybean hairy roots, we analysed the cellular localization of the overexpressed Rhg1‐GmAAT (Figs 1j and S2, see Supporting Information). For nucleus staining, the reagent 4,6‐diamidino‐2‐phenylindole (DAPI) (Sigma, USA) was used. The green signal of P_35S_‐GmAAT‐GFP overlapped the blue signal of DAPI (Figs S1j and S2a). To verify the plasma membrane localization, we induced plasmolysis of onion epidermal cells expressing P_35S_‐GmAAT‐GFP by treatment with a high concentration of NaCl solution. P_35S_‐GmAAT‐GFP was observed in the plasma membrane after plasmolysis (Fig. S2a). Therefore, we speculated that P_35S_‐GmAAT‐GFP was located in both the nucleus and plasma membrane. As there was no clear difference between the control P_35S_‐GFP and fusion protein P_35S_‐GmAAT‐GFP images, P_35S_‐GmAAT‐GFP might also exist in the cytoplasm.

### Glutamate may be the substrate of Rhg1‐GmAAT *in vivo*

**Figure 2:**
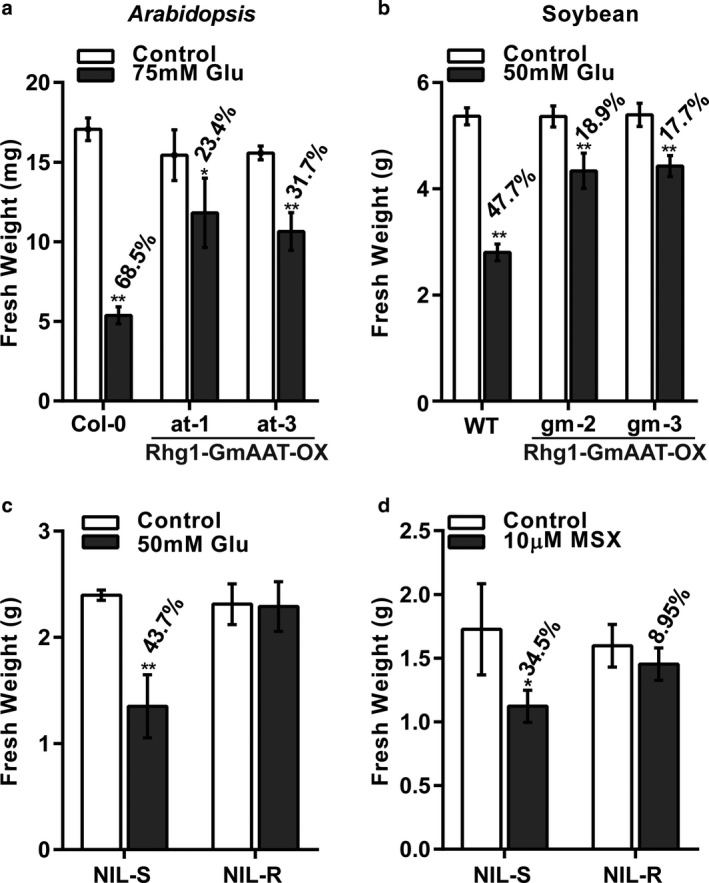
Growth of plants subjected to toxic levels of glutamic acid (Glu) or the toxic Glu analogue *N*‐methyl sulfoximine (MSX). (a) Col‐0 and Rhg1‐GmAAT‐OX (Rhg1‐GmAAT‐overexpressing)* Arabidopsis* lines were grown for 3 weeks on half‐strength Murashige and Skoog medium containing 75 mm Glu. (b) The wild‐type (cultivar Tianlong 1) and Rhg1‐GmAAT‐OX soybean lines were grown for 3 days in quarter‐strength Murashige and Skoog liquid medium containing 50 mm Glu. (c, d) Two soybean near‐isogenic lines (NIL‐S and NIL‐R) were grown for 3 days in quarter‐strength Murashige and Skoog liquid medium containing either 50 mm Glu (c) or 10 μm MSX (d). Fresh weights were measured. The values are the means ± standard deviations (SDs) (*n* = 4). *0.01 < *P* < 0.05, **P < 0.01 (multiple *t*‐test followed by the Holm–Sidak *post hoc* test). The values above the columns provide the percentage of inhibition compared with that of untreated plants. WT, wild‐type.

To analyse the substrate of Rhg1‐GmAAT in plants, we performed a growth inhibition assay which involved the evaluation of the tolerance of plants to excess amounts of different amino acids. Exogenous treatment with toxic level of amino acids caused a feedback inhibition of the biosynthesis of the shared precursors of other amino acids *in vivo*, resulting in stunted plant growth (Less and Galili, 2008). Two transgenic *Arabidopsis* lines, at‐1 and at‐3 (Fig. S3a, see Supporting Information), overexpressing Rhg1‐GmAAT (Rhg1‐GmAAT‐OX), and a control line ecotype Columbia‐0 (Col‐0), were sown on medium supplemented with excess amounts of different amino acids, as described by Lee *et al*. (2007). The Rhg1‐GmAAT‐OX lines grew better than the Col‐0 line only on medium containing Glu (Fig. 2a). Under other experimental conditions, all plants exhibited severely stunted growth (Fig. S4, see Supporting Information). Tolerance to the treatment of excess Glu suggested that *Rhg1‐GmAAT* might specifically impact glutamate transportation in plants.

To further investigate the function of *Rhg1‐GmAAT* in soybean, two homozygous transgenic soybean Rhg1‐GmAAT‐OX lines (gm‐2 and gm‐3) in the cultivar Tianlong 1 background (Fig. S3b) were generated, and a growth inhibition assay was also conducted. Toxic levels of Glu, Gln, Asp and glycine (Gly) were used to treat the WT (cultivar Tianlong 1) and Rhg1‐GmAAT‐OX plants. At 72 h after treatment, the WT plants were wilted and their fresh weight had decreased by 47.7%. In contrast, the fresh weight of Rhg1‐GmAAT‐OX plants decreased by only 18.9% and 17.7% (Fig. 2b). No difference between the WT and transgenic lines was observed in response to Gln, Asp and Gly treatments (Fig. S5, see Supporting Information). The growth of all plants was severely affected. Obviously, in soybean, overexpression of *Rhg1‐GmAAT* enhances the tolerance of seedlings specifically to excess amounts of Glu.

The more tolerant phenotype could possibly be caused by the constitutive overexpression of *Rhg1‐GmAAT* rather than by its true function *in viv*o. To check this possibility, we compared the tolerance to Glu of a pair of near‐isogenic lines (NILs) for *Rhg1*, NIL‐R and NIL‐S. *Rhg1‐GmAAT* in the NIL‐R lines was expressed three‐ to four‐fold more strongly than in the NIL‐S lines (Fig. S3c). NIL‐S and NIL‐R were susceptible and resistant to SCN, respectively (Fig. S6, see Supporting Information). After being treated with 50 mm Glu, the fresh weights of NIL‐S plants were significantly decreased (*P* = 4.41E‐04). In comparison, the fresh weights of NIL‐R plants were nearly unchanged (Fig. 2c). To further confirm the natural function of Rhg1‐GmAAT, NIL‐S and NIL‐R plants were treated with 10 μm methionine sulfoximine (MSX), which is a toxic analogue of Glu (Rawat *et al*., 1999); at low levels (1 μm), MSX is toxic to *Arabidopsis* Col‐0 (Perchlik *et al*., 2014). The fresh weights of MSX‐treated NIL‐R plants decreased by 8.95%, whereas that of MSX‐treated NIL‐S plants deceased by 34.5% (Fig. 2d), indicating that NIL‐R plants are more tolerant than NIL‐S plants to MSX. All of these observations were similar to those of transgenic *Arabidopsis* and soybean plants, suggesting that the natural overexpression of *Rhg1‐GmAAT* in plants may provide tolerance to toxic levels of exogenous Glu.

**Figure 3:**
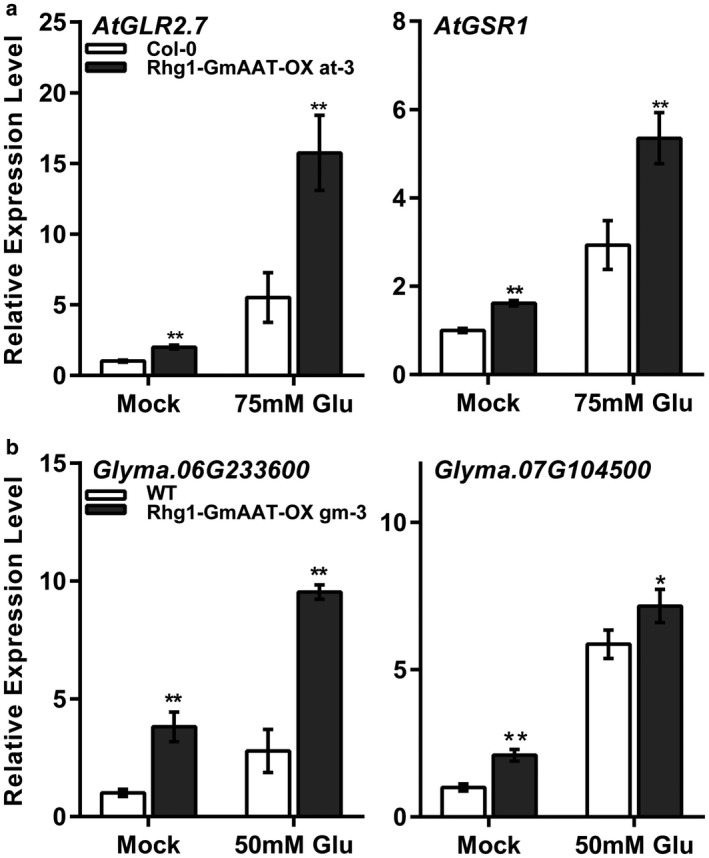
Expression of the glutamate receptor‐like genes and glutamine synthetase genes in wild‐type and Rhg1‐GmAAT‐overexpressing (Rhg1‐GmAAT‐OX) lines in *Arabidopsis* and soybean. (a) *AtGLR2.7* and *AtGSR1* expressed in the roots of 75 mm glutamic acid (Glu) ‐treated Col‐0 and Rhg1‐GmAAT‐OX line at‐3. The expression levels were normalized to *ATACT2.* (b) *Glyma.06G233600* and *Glyma.07G104500* expressed in the roots of 50 mm Glu‐treated wild‐type (cultivar Tianlong 1) and Rhg1‐GmAAT‐OX line gm‐3. The expression levels were normalized to *SKIP16*. The values are the means ± standard deviations (SDs) (*n* = 3). Asterisks indicate a statistically significant difference of Rhg1‐GmAAT‐OX lines compared with the wild‐type under the same conditions. *0.01 < *P* < 0.05, **P < 0.01; multiple *t*‐test followed by the Holm–Sidak *post hoc* test. WT, wild‐type.

The expression of glutamate receptor‐like genes and Gln synthetase genes is sensitive to glutamate uptake (Masclaux‐Daubresse, 2006). In *Arabidopsis*, expression of the glutamate receptor gene *AtGLR2.7* (*AT2G29120*) and the Gln synthesis gene *AtGSR1* (*AT5G37600)* was analysed in roots of 2‐week‐old Col‐0 and Rhg1‐GmAAT‐OX line at‐3 growing on medium with or without 75 mm Glu. In soybean, the expression levels of the glutamate receptor‐like gene *Glyma.06G233600 *and Gln synthetase PR‐2 gene *Glyma.07G104500 *were detected in 50 mm Glu‐treated soybean roots of the WT (cultivar Tianlong 1) and Rhg1‐GmAAT‐OX line gm‐3 at 2 days post‐treatment. As expected, the treatment of Glu induced the expression of these sensor genes (Fig. 3) in both plant species. Compared with WT plants, Rhg1‐GmAAT‐OX plants demonstrated higher expression levels of the sensor genes under conditions with or without a toxic level of Glu (*P* < 0.05). These results indicate that the overexpression of *Rhg1‐GmAAT* might enhance the accumulation of glutamate in roots.

### Overexpression of *Rhg1‐GmAAT* increases the transportation of glutamate from shoots to roots in soybean

**Figure 4:**
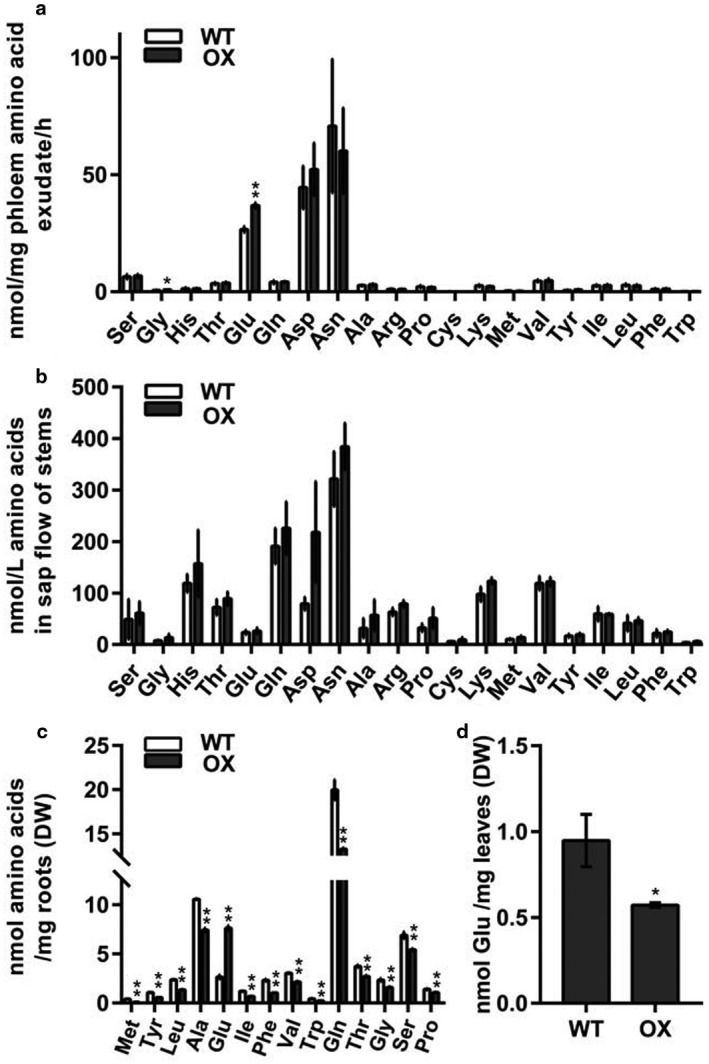
Amino acid contents in the leaf phloem exudates, root xylem exudates, leaves and roots of the wild‐type (cultivar Tianlong 1) and Rhg1‐GmAAT‐overexpressing (Rhg1‐GmAAT‐OX) line gm‐3. (a) Amino acid analysis of the phloem exudates of leaves. (b) Amino acid analysis of the sap flow of stems (*n* = 4). (c) Free amino acid contents of 21‐day‐old roots. (d) Free glutamic acid (Glu) contents of 21‐day‐old leaves. Leaf measurements were calculated using the dry weight (DW) in (a) and (d). Root measurements were calculated using the DW in (c). Four plants constituted a sample; three samples per line were used for (c) and (d). The values are the means ± standard deviations (SDs) (*n* = 3). *0.01 < *P* < 0.05, **P < 0.01 (multiple *t*‐test followed by the Holm–Sidak *post hoc* test). The experiments were repeated two or three times, each producing similar results. WT, wild‐type; OX, Rhg1‐GmAAT‐OX line gm‐3.

Because the promoter of *Rhg1‐GmAAT* was active in the root vascular tissue, the expression of *Rhg1‐GmAAT* was expected to affect amino acid transport. Excess amino acids synthesized in the leaves are exported via the phloem (Tegeder *et al*., 2012). To investigate the effects of increased expression of *Rhg1‐GmAAT* on phloem amino acid transport, we used high‐performance liquid chromatography coupled to tandem mass spectrometry (HPLC‐MS/MS) to examine the amino acid content in the phloem exudates of Rhg1‐GmAAT‐OX and WT plants (cultivar Tianlong 1). Compared with WT plants, Rhg1‐GmAAT‐OX plants exhibited relatively higher amounts of free Glu (*P* = 0.003) and Gly (*P* = 0.025) in the leaf phloem (Fig. 4a), suggesting that the transportation of glutamate and Gly from the shoots to the roots might be enhanced by the overexpression of *Rhg1‐GmAAT*. Amino acids in the xylem are mainly derived from the roots (Ortiz‐Lopez *et al*., 2002). To investigate the transportation of amino acids from the roots to the shoots, we compared the free amino acid contents in the root xylem sap between Rhg1‐GmAAT‐OX and WT (cultivar Tianlong 1) plants, and detected similar amounts of individual amino acids between the two (Fig. 4b), suggesting that the transportation of glutamate from the roots to the shoots was not affected by the overexpression of *Rhg1‐GmAAT*. The above results imply that Rhg1‐GmAAT may positively regulate the transportation of glutamate from the shoots to the roots.

By using HPLC‐MS/MS, we then compared the amounts of free amino acids between the roots and leaves. Compared with the leaves, the roots of Rhg1‐GmAAT‐OX plants presented a 2.88‐fold increase in Glu content and a 21.4%–75.3% decrease in several amino acid contents, including those of Met, tyrosine (Tyr), leucine (Leu), Ala, isoleucine (Ile), phenylalanine (Phe), valine (Val), Trp, Gln, threonine (Thr), Gly and serine (Ser). No significant differences were observed in the total amounts of free amino acids, which were obtained by summing the individual amino acids (Fig. 4c; Table S1, see Supporting Information). In contrast, the Glu contents of the Rhg1‐GmAAT‐OX leaves were reduced by 39.7% compared with those of WT. However, no significant differences were observed in the other measured individual free amino acids (Fig. 4d; Table S1). The data confirm that the overexpression of *Rhg1‐GmAAT* enhances the transportation of glutamate from the shoots to the roots and consequently increases the glutamate content in the roots.

### Overexpression of Rhg1‐GmAAT activates the JA pathway in soybean

**Figure 5:**
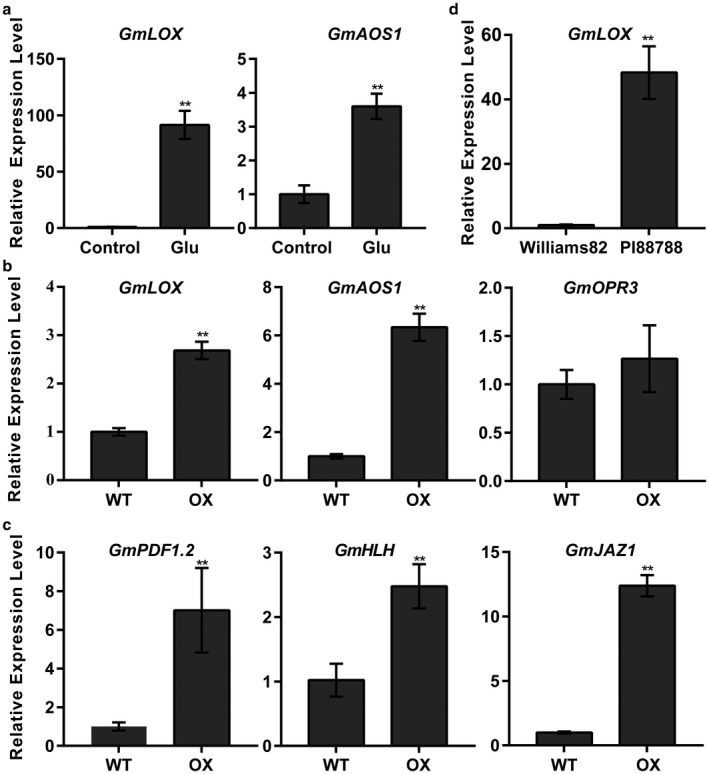
Expression of a set of jasmonic acid (JA) biosynthesis and JA‐responsive genes. (a) JA biosynthesis genes expressed in the roots of glutamic acid (Glu) ‐treated soybean. Roots were treated with quarter‐strength Murashige and Skoog medium that either lacked nitrogen (control) or was supplemented with 5 mm Glu for 24 h. JA biosynthesis genes (b) and JA‐responsive genes (c) expressed in the roots of the wild‐type (cultivar Tianlong 1) and Rhg1‐GmAAT‐overexpressing (Rhg1‐GmAAT‐OX) line gm‐3. (d) JA biosynthesis genes expressed in the roots of Williams 82 and PI88788. The expression levels of the selected genes were assayed by quantitative reverse transcription‐polymerase chain reaction (qRT‐PCR) and were normalized to *SKIP16*. The values are the means ± standard deviations (SDs) (*n* = 3). **0.01 < *P* < 0.05, **P < 0.01 (multiple *t*‐test followed by the Holm–Sidak *post hoc* test). WT, wild‐type; OX, Rhg1‐GmAAT‐OX line gm‐3.

Endogenous and exogenous amino acids are key players in plant defence against pathogens (Seifi *et al*., 2013). Previous studies in citrus have suggested that the accumulation of Glu induces the JA pathway and subsequently activates the systemic defence response to herbivore attack (Agut *et al*., 2016). JA synthesis starts with linolenic acid, which is converted to OPDA via the octadecanoid pathway; this conversion occurs via the sequential action of the chloroplast‐localized enzymes lipoxygenase (LOX), allene oxide synthase (AOS) and allene oxide cyclase (AOC), after which OPDA is then converted to JA in the peroxisome by OPDA reductase3 (OPR3) (Taki *et al*., 2005; Wasternack and Hause, 2013). GmLOX (type II 13‐LOX) (Song *et al*., 2016) and GmAOS1 (Kongrit *et al*., 2007) serve critical functions in JA biosynthesis. To determine whether the JA pathway can be induced by enhanced applications of Glu in soybean, we treated the roots of soybean seedlings with 5 mm Glu for 24 h and used real‐time qRT‐PCR to measure the expression of *GmLOX* (*Glyma.03G264300*) and *GmAOS1 *(*Glyma.14G078600*). The results showed that, compared with those in control seedlings, the expression levels of *GmLOX* and *GmAOS1* in treated seedlings increased by up to approximately 91‐fold and four‐fold, respectively (Fig. 5a). These observations indicate that JA biosynthesis is activated in response to exogenous Glu applications to soybean roots. Considering the increased accumulation of free Glu in the roots of Rhg1‐GmAAT‐OX plants, we inferred that JA biosynthesis might be activated *in vivo* in Rhg1‐GmAAT‐OX plants.

To support this inference, we compared the transcriptomes between the roots of 3‐week‐old Rhg1‐GmAAT‐OX line gm‐3 and WT (cultivar Tianlong 1) plants. A total of 1607 differentially expressed genes (DEGs) with two‐fold cut‐off values were identified [false discovery rate (FDR) < 0.05]. Enrichment analysis of the Kyoto Encyclopedia of Genes and Genomes (KEGG) suggested that the plant–pathogen interaction pathway was the most enriched pathway for these DEGs in Rhg1‐GmAAT‐OX plants (Fig. S7, see Supporting Information). In addition, a set of up‐regulated JA‐related genes was identified in Rhg1‐GmAAT‐OX plants (Table S2, see Supporting Information). The expression levels of the JA biosynthesis genes (*GmLOX*, *GmAOS1* and *GmOPR3*) and JA signalling pathway genes [*GmPDF1.2 *(*Glyma.18G027700*), *GmJAZ1 *(*Glyma.11G038600*) and *GmbHLH35 *(*Glyma.17G058600*)] were detected and verified by qRT‐PCR (Fig. 5b,c)*.* These results suggest that the JA pathway may be up‐regulated by the overexpression of *Rhg1‐GmAAT* in soybean.

**Figure 6:**
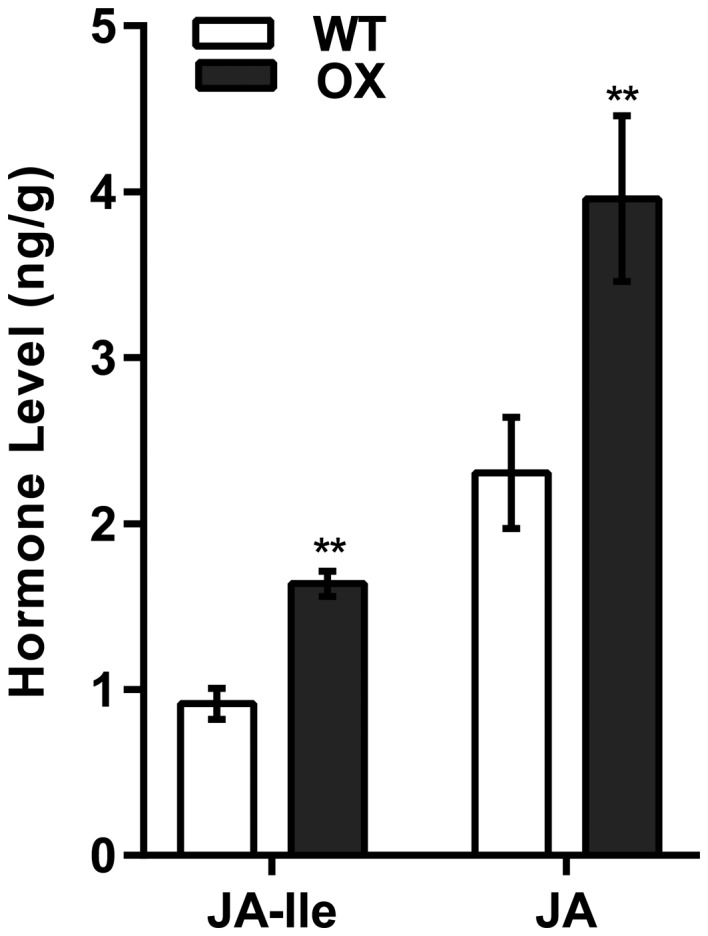
Jasmonic acid (JA) content in the roots of the wild‐type (cultivar Tianlong 1) and Rhg1‐GmAAT‐overexpressing (Rhg1‐GmAAT‐OX) line gm‐3. Seedlings were cultured in quarter‐strength Murashige and Skoog medium for 4 weeks. Endogenous JA and jasmonoyl isoleucine (JA‐Ile) of roots were quantified using high‐performance liquid chromatography coupled to tandem mass spectrometry. The values are the means ± standard deviations (SDs) (*n* = 3). **0.01 < *P* < 0.05, **P < 0.01 (multiple *t*‐test followed by the Holm–Sidak *post hoc* test). WT, wild‐type; OX, Rhg1‐GmAAT‐OX line gm‐3.

As the JA pathway was up‐regulated by the overexpression of *Rhg1‐GmAAT*, we performed a metabolite analysis of the roots of transgenic plants to determine whether the overexpression of Rhg1‐GmAAT induced the JA content. A total of 545 compounds were detected in the metabolite assay; 36 significant differences occurred, 29 of which were suppressed and seven of which were induced. Four kinds of plant hormones changed significantly; JA was the only induced hormone (Table S3, see Supporting Information). To verify these results, an absolute quantitative technique was used to analyse the JA content in the roots of both the WT (cultivar Tianlong 1) and Rhg1‐GmAAT‐OX soybean plants. Compared with WT roots, the JA and JA‐Ile contents in the Rhg1‐GmAAT‐OX roots significantly increased by up to 1.717‐fold (*P* = 1.92E‐06) and 1.789‐fold (*P* = 5.22E‐07), respectively (Fig. 6). MeJA was not detected in any of the samples. These results support the hypothesis that Rhg1‐GmAAT positively affects the accumulation of JA.

To study the changes in the JA biosynthesis pathway in populations that exhibit a naturally high expression of *Rhg1‐GmAAT*, the transcripts of *Rhg1‐GmAAT* and the JA biosynthesis gene *GmLOX* were measured in Williams 82 (PI518671) and PI88788, which contain one and eight copies of *Rhg1*, respectively (Lee *et al*., 2015). Consistent with the copy number results, PI88788 exhibited progressively higher transcript levels of* Rhg1‐GmAAT* and* GmLOX* than did Williams 82 (Figs 5d and S3d). Furthermore, JA content was compared between a bulk (Bulk‐R) of naturally resistant soybean varieties (PI437655, PI495017C, PI209332, PI438503A and PI467312) and a bulk (Bulk‐S) of susceptible soybean varieties [Hutcheson (PI518664), Magellan (PI595362) and Williams 82]. The content of JA in Bulk‐R was 1.84 times (*P* = 1.37E‐07) that of JA in Bulk‐S. In detail, PI88788 contained 2.92 times (*P* = 2.17E‐10) more JA than Hutcheson (Fig. S8, see Supporting Information). These observations accord with the hypothesis that JA is involved in SCN resistance.

### Inhibition of the JA pathway reduces the resistance to SCN in Rhg1‐containing soybean germplasm

**Figure 7:**
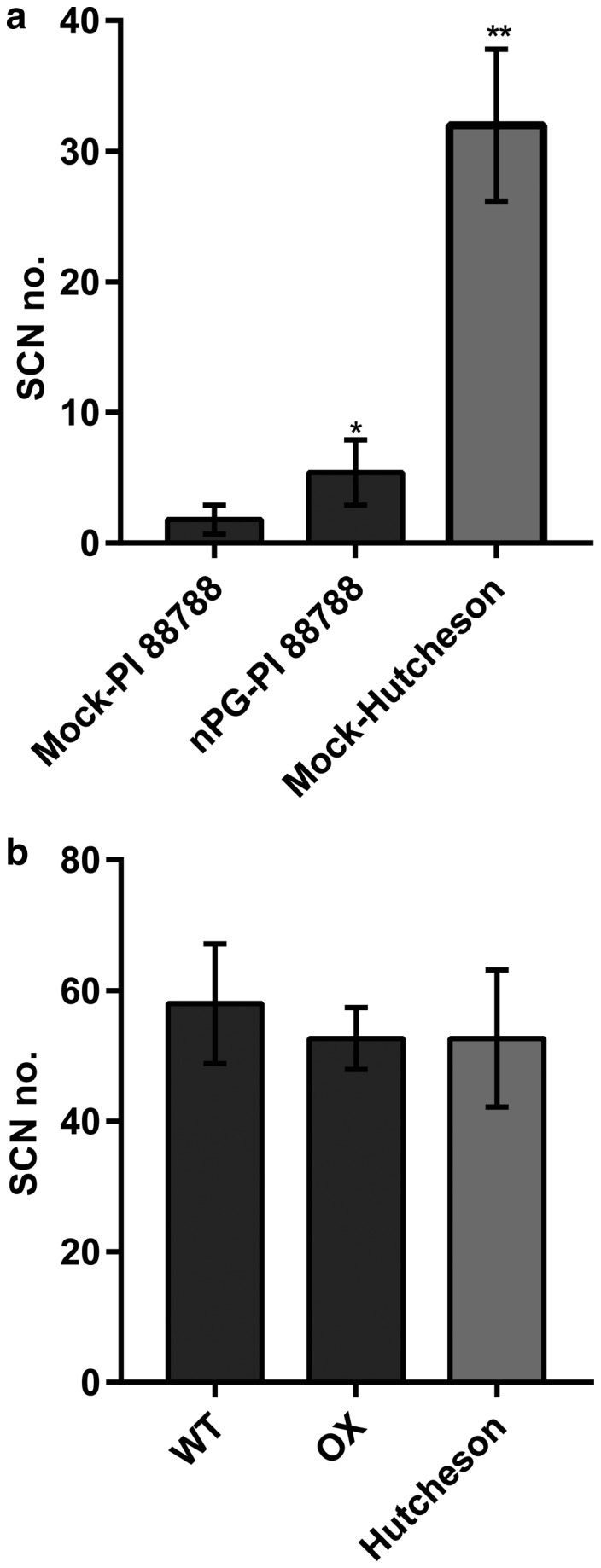
Responses of n‐propyl gallate (nPG)‐treated PI88788 and Rhg1‐GmAAT‐overexpressing (Rhg1‐GmAAT‐OX) line to soybean cyst nematodes (SCNs). (a) PI88788 and Hutcheson soybean seedlings were pretreated with either water containing 0.02% ethanol (mock) or a 100 μm nPG solution for 3 days prior to inoculation with nematodes. The values are the means ± standard deviations (SDs) (*n* = 6). Asterisks indicate a statistically significant difference of nPG‐treated PI88788 and mock‐treated Hutcheson compared with mock‐treated PI88788. *0.01 < *P* < 0.05, **P < 0.01 (multiple *t*‐test followed by the Holm–Sidak *post hoc* test). (b) The wild‐type (cultivar Tianlong 1), Rhg1‐GmAAT‐OX line gm‐3 and Hutcheson soybean seedlings were inoculated with nematodes. The values are the means ± SDs (*n* = 6). Female cysts were quantified after 30 days. The experiments were repeated at least three times, each producing similar results. WT, wild‐type; OX, Rhg1‐GmAAT‐OX line gm‐3.

As the JA pathway was activated in naturally resistant germplasm, we sought to confirm the involvement of the JA pathway in conferring resistance to SCN. As such, by application of the JA biosynthesis inhibitor n‐propyl gallate (nPG) (Bruinsma *et al*., 2010; Pena‐Cortés *et al*., 1993) to inhibit JA biosynthesis (Fig. S9, see Supporting Information), the changes in resistance to SCN in PI88788 were evaluated. As shown in Fig. 7a, the average numbers of cysts in mock‐treated Hutcheson, mock‐treated PI88788 and nPG‐treated PI88788 were 32, 1.8 and 5.4, respectively. nPG treatment increased the cysts in PI88788 from 5.6% to 16.9%, with the number of cysts on Hutcheson being 100% (*P* = 0.02). As expected, inhibition of JA makes soybean more susceptible (Kammerhofer *et al*., 2015; Li *et al*., 2018; Zhang *et al*., 2017).

No difference in resistance to SCN was observed between Rhg1‐GmAAT‐OX plants and cultivar Tianlong 1 control plants (Fig. 7b), which indicated that overexpression of *Rhg1‐GmAAT* alone in soybean could not provide resistance to SCN. These results were consistent with those in previous reports by Cook *et al*. (2012), in that three genes rather than one were responsible for resistance to SCN at *Rhg1*.

## Discussion

In this study, we explored the function of *Rhg1‐GmAAT*, one of three genes at *Rhg1*, which is a major quantitative trait locus involved in resistance to SCNs (Cook *et al*., 2012). Based on analyses of sequence and expression patterns, *Rhg1‐GmAAT* was predicted to encode an amino acid transporter, which was expressed mostly in the vasculature (Fig. 1b–i). Subcellular localization revealed that Rhg1‐GmAAT was located in the plasma membrane and nucleus, and might also exist in the cytoplasm (Figs 1j and S2). Some transporters located in the plasmalemma are thought to be involved in amino acid absorption and distribution (Ladwig *et al*., 2012; Lee *et al*., 2007; Su *et al*., 2004; Tegeder *et al*., 2012). A growth inhibition assay was performed to evaluate the tolerance of plants to toxic levels of amino acids, and found that Rhg1‐GmAAT might impact the transportation of glutamate (Figs 2, 3, S4 and S5). In addition to the role of Rhg1‐GmAAT as an amino acid transporter, the nuclear localization of Rhg1‐GmAAT suggested that this protein might have other functions. For example, when localized in the plasma membrane, the mammalian amino acid transporter PAT1 (SLC36A1) functions in amino acid transportation, but, when localized in the nucleus, PAT1 functions as an amino acid sensor (Chen *et al*., 2003; Jensen *et al*., 2014). Whether Rhg1‐GmAAT functions as an amino acid sensor is an interesting question to be answered.

The measurements of free Glu contents in the phloem exudates and xylem sap showed that overexpression of *Rhg1‐GmAAT* could enhance the long‐distance transportation of glutamate from the shoots to the roots without affecting the transportation from the roots to the shoots; this phenomenon was further demonstrated by the increased content of free Glu in the roots and the decreased content in the shoots in transgenic Rhg1‐GmAAT‐OX plants (Fig. 5; Table S1). These results also support the hypothesis that Rhg1‐GmAAT functions as an amino acid transporter *in vivo*. As a signalling molecule, glutamate metabolism can regulate plant hormone pathways (Kadotani *et al*., 2016; Liu *et al*., 2010; Seifi *et al*., 2013). Kadotani *et al*. (2016) reported that exogenous Glu induced blast resistance partially dependent on salicylic acid (SA) signalling. Agut *et al*. (2016) reported that, in *Citrus aurantium* (sour orange), the roots exported Glu to the shoots to trigger the transcription of glutamate receptors in order to induce JA biosynthesis to increase resistance against spider mites. In this study, the treatment of soybean roots with Glu significantly activated the transcription of JA biosynthesis genes (Fig. 5a), which suggested that the increased Glu content might stimulate JA biosynthesis *in vivo* in soybean. We then performed RNA‐sequencing (RNA‐Seq) and metabolite analyses of the roots of transgenic Rhg1‐GmAAT‐OX soybean plants; the results confirmed our speculation (Figs 5, 6, S7; Tables S2 and S3). JA has been implicated in resistance to nematodes (Heil and Ton, 2008; Schilmiller and Howe, 2005). In soybean, the majority of JA pathway components are suppressed during compatible soybean SCN infection (Ithal *et al*., 2007a). The up‐regulation of soybean *LOX* genes, which are key for JA biosynthesis, has been reported in syncytia induced by SCN on Peking and PI88788 plants (Klink *et al*., 2009, 2010). Matthews *et al*. (2014) overexpressed three *Arabidopsis *genes (*AtAOS*, *AtAOC*, *AtJAR*) involved in JA/JA‐Ile production in soybean, which improved resistance to SCN (Lin *et al*., 2013). To confirm whether the JA pathway is involved in *Rhg1*‐mediated resistance to SCN, we used a JA biosynthesis inhibitor, nPG, to inhibit the JA pathway in the naturally SCN‐resistant soybean accession PI88788. Compared with the mock‐treated control roots, the nPG‐treated roots presented significantly more female cysts (Fig. 7a), indicative of a role for the JA pathway in *Rhg1*‐mediated resistance to SCN.

Recently, the JA pathway has been discovered to consist of two antagonistic branches: one branch is characterized by the basic helix‐loop‐helix leucine zipper transcription factor (TF) MYC2, whereas the other is characterized by the apetala2/ethylene response factor (AP2/ERF) domain TF ORA59 (Verhage *et al*., 2011). The MYC2 branch can be induced by damage‐triggered immunity responses and is marked by the strong induction of the marker gene *vegetative storage protein 2* (*VSP2*) in *Arabidopsis*. In *Arabidopsis*, the ERF branch is induced by oral secretions from *Pieris rapae* larvae; *PDF1.2* is the marker gene (Verhage *et al*., 2011). During the early stages of nematode parasitism in *Arabidopsis*, phosphatase AP2C1 negatively regulates the mitogen‐activated protein kinases (MAPKs) MPK3 and MPK6; these kinases are two positive regulators of plant defence responses and control ET and JA biosynthesis (Sidonskaya *et al*., 2016). The enhanced susceptibility of *mpk3 *and *mpk6* plants to sugar beet cyst nematode (*Heterodera schachtii*) indicates a positive role of stress‐activated MAPKs in plant immunity against nematodes (Sidonskaya *et al*., 2016). In addition, the up‐regulation of VSP2, a marker for the MYC2 branch of the JA pathway, occurs on infection by the SCN *Heterodera glycines* in both *Arabidopsis* and soybean roots (Afzal *et al*., 2009; Puthoff *et al*., 2003); this up‐regulation is due to damage‐triggered immunity responses induced by the intracellular migration of cyst nematodes before the establishment of a permanent feeding site. In our study, the expression of *Rhg1‐GmAAT* induced the transcription of *GmMPK3* (*Glyma.12G073000* and *Glyma.U021800*) and *GmPDF1.2*, a marker of the ERF branch of the JA pathway, but suppressed the expression of *GmVSPβ *(Table S2). These results imply that Rhg1‐GmAAT might regulate the biosynthesis of JA via the MPK3 and MPK6 pathways, and that the ERF branch of the JA pathway might play a role in the resistance to SCN.

However, our metabolite profiling results were opposite to the established notion that JA induces other specialized metabolites. Most components were suppressed, not induced. This might be related to the up‐regulation of the *JAZ* genes, repressors of JA signalling, in Rhg1‐GmAAT‐OX plants (Table S2). These results suggest that Rhg1‐GmAAT could mediate JA signalling. Moreover, the fine regulation of the *Rhg1‐GmAAT* gene on the JA biosynthesis and signalling pathway may contribute to the small number of induced compounds. Another reason might be that the method we used to conduct the metabolite profiling was not capable of detecting all kinds of metabolites, especially some specialized secondary metabolites in plants. Therefore, some JA‐induced metabolites were unable to be detected, which caused the small number of JA‐induced metabolites in this study. More specific mechanisms need to be further studied in the future.

At *Rhg1*, three tightly linked genes have been shown to contribute to resistance to SCN (Cook *et al*., 2012). These include an amino acid transporter gene (*Glyma.18G022400*), an α‐SNAP gene (*Glyma.18G022500*) and a WI12 gene (*Glyma.18G022700*) (Cook *et al*., 2012). These three genes must be simultaneously overexpressed to provide resistance to SCN (Cook *et al*., 2012). Our results also showed that overexpression of *Rhg1‐GmAAT* alone does not influence resistance to SCN (Fig. 6b). Until now, how these three genes coordinately function to provide resistance to SCN has remained unknown. By analysis of the function of each gene individually, scientists have attempted to answer this question. The function of the α‐SNAPs in *Rhg1*‐mediated resistance has been elucidated (Bayless *et al*., 2016; Liu *et al*., 2017). The *Rhg1* resistance‐type α‐SNAPs interrupt NSF function and vesicle trafficking, which results in cytotoxicity at sedentary feeding sites (Bayless *et al*., 2016). Our results showed that, by activating the JA pathway, Rhg1‐GmAAT might be involved in the transport of glutamate *in vivo* and play a role in the resistance to SCN. No studies on the function of Rhg1‐GmWI12 in the resistance to SCN have been conducted. The WI12 gene in ice plants (*Mesembryanthemum crystallinum*) is involved in cell wall modification at wound sites and is induced by JA (Yen *et al*., 2001). Our RNA‐Seq results showed that overexpression of *Rhg1‐GmAAT* increased the transcription of Rhg1‐GmWI12 by 1.99‐fold (Table S2). In addition, Rhg1‐GmWI12 could also be induced by JA in soybean (Fig. S10, see Supporting Information). We speculated that Rhg1‐GmWI12 might play a role in the intracellular migration of cyst nematodes, where the responses of plants to wounding mainly occurred. The three responsible genes at *Rhg1 *might have different or interrelated functions during the processes of nematode invasion and colonization. Once WI12 is elucidated, the coordination mechanism of these three genes with respect to resistance to SCN needs to be investigated to clarify the whole process concerning *Rhg1*‐mediated resistance to SCN in soybean.

In summary, our results showed that Rhg1‐GmAAT might be involved in the transport of glutamate *in vivo*. Overexpression of *Rhg1‐GmAAT* increased the transportation of glutamate from the shoots to the roots, resulting in both the accumulation of free Glu in the roots and the up‐regulation of the JA pathway, which contributed to resistance to SCN in an *Rhg1*‐containing resistant germplasm. Our results could be helpful for an understanding of the whole *Rhg1*‐mediated mechanism of resistance to SCN in soybean.

## Experimental Details

### Plant material and growth conditions

Plants of the soybean cultivar Tianlong 1, PI88788, transgenic lines and NILs were obtained from the Oil Crops Research Institute, Chinese Academy of Agricultural Sciences, Wuhan, China. The NILs (NIL‐R and NIL‐S) were derived from a cross of PI437654 and Williams 82 through marker‐assisted selection. The resistant soybean accession PI437654, which contains low copies of *Rhg1*‐a and *Rhg4*, was backcrossed four times to the susceptible accession Williams 82 to produce the BC_4_F_2_ progenies. The BC_4_F_2_ plants, which were homozygous at *Rhg4* but heterozygous at *Rhg1*, were self‐pollinated to produce the BC_4_F_2:3_ individuals. From these individuals, the NILs of *Rhg1* were developed, whose phenotypes were also confirmed in the glasshouse by evaluation of their resistance to SCN. The NILs (NIL‐R and NIL‐S) were derived from a cross between the resistance source PI437654 and the susceptible Williams 82. Soybean (*Glycine max*) plants were cultivated in the glasshouse at 25 °C under a 16‐h light/8‐h dark photoperiod. *Arabidopsis thaliana* Col‐0 and transgenic plants were grown in a growth chamber under an 8‐h light/16‐h dark photoperiod and 75% relative humidity at 22 °C.

### Growth of *Arabidopsis* and soybean plants on medium containing excess amounts of amino acids or toxic Glu analogues

*Arabidopsis* Col‐0 (representing WT) and Rhg1‐GmAAT‐OX lines were sown on half‐strength Murashige and Skoog medium supplemented with excess amounts of various amino acids [2 mm Tyr or Lys; 4 mm Leu or Met; 6 mm Val; 10 mm Phe, Ile, Ser, Thr or Trp; 20 mm His; 25 mm cysteine (Cys) or Gly; 50 mm asparagine (Asn) or Asp; and 100 mm Gln, Ala, proline (Pro), Arg or Glu] and grown for 21 days.

Soybean cultivar Tianlong 1 (representing WT), transgenic overexpressed Rhg1‐GmAAT‐OX and NIL (NIL‐R and NIL‐S) plants were cultured in quarter‐strength Murashige and Skoog medium supplemented with 50 mM Glu, 10 μm MSX (a toxic Glu analogue), 50 mm Asp, 75 mm Gln and 25 mm Gly, and then grown for 3 days. These studies were repeated at least three times, during which the plants were imaged. The fresh weights of whole plants were determined for three experiments by measuring four to six plants.

### DNA constructs

To overexpress *Rhg1‐GmAAT *(*Glyma.18g022400*), the full‐length coding sequence of soybean Williams 82 cDNA was amplified and cloned into a pGWC entry vector (Chen *et al*., 2006). The constructed entry vector was recombined into a pB2GW7 plant transformation vector (Karimi *et al*., 2002) using the LR recombinase reaction (Invitrogen, USA). To visualize GFP in tobacco epidermal cells and soybean hairy roots, *Rhg1‐GmAAT *cDNA was cloned into a pEGAD‐GFP vector using the *Age*I restriction site, generating P_35S_‐GmAAT‐GFP. To generate *P_GmAAT_‐GUS* chimeric genes, the 1.6‐kb promoter region of *Rhg1‐GmAAT *from soybean Williams 82 genomic DNA was amplified and cloned upstream of the GUS reporter gene within the plant binary vector pCX‐GUS‐P (Chen *et al*., 2009). Unless otherwise specified, all constructs were generated by a sequence‐independent, ligation‐free method using a ClonExpress II One Step Cloning Kit (Vazyme, China).

The plant transformation constructs were introduced into *Agrobacterium tumefaciens* GV3101 and EHA105 strains by the freeze–thaw method. The GV3101 strains were used to transform *Arabidopsis* Col‐0 plants by the floral dip method (Clough and Bent, 1998). The EHA105 strains were used to transform soybean cultivar Tianlong 1 plants via the *Agrobacterium*‐mediated transformation method (Paz *et al*., 2006). Transformed lines were selected on soil by spraying with a 1 : 1000 dilution of Basta (Bayer CropScience, Germany).

### Semi‐quantitative and quantitative real‐time RT‐PCR

The total RNA was extracted from plants with TRIzol reagent (Invitrogen). A sample containing 2 μg of total RNA was treated with DNase I (Invitrogen) and reverse transcribed with M‐MLV Reverse Transcriptase (Promega, USA). Semi‐quantitative PCR was then performed using PCR Master Mix (MBI Fermentas, Canada). qRT‐PCR was performed using Takara SYBR Premix Ex Taq following the manufacturer’s instructions (Takara, Japan) in an ABI Q3 or Q5 qRT‐PCR system (Applied Biosystems, USA). Three biological repeats were used and each of three separate cDNA samples for the same condition was used for the statistical analysis. *ATACT 2* and *SKIP16* were used as *Arabidopsis* and soybean internal reference genes, respectively. Normalized fold expression levels were calculated using the –ΔΔC(t) method. Unless otherwise indicated, the results are relative to those of untreated WT plants, which were set to a relative value of unity. The primers used are listed in Table S4 (see Supporting Information). The data show the representative results of three biological replicates.

### Amino acid quantification

Samples were ground to a powder in liquid N_2_ and subsequently dissolved in ultrapure water. Derivatization was performed with an MSLAB‐45+AA kit (MSLAB, China). HPLC‐MS/MS analyses were performed using an Ultimate 3000 HPLC system (Dian, USA) connected to an API 3200Q TRAP liquid chromatograph coupled to a tandem mass spectrometry (LC/MS/MS) system (Applied Biosystems), in accordance with the procedures described by Spitzner *et al*. (2008), by MSLAB. Estimation was performed in positive mode by multiple reaction monitoring (MRM) and a scan time of 20 min. The first transition was used for quantification; the other was used for confirmation. An equation developed from a five‐point calibration was used as a standard.

### Leaf phloem exudate and root xylem exudate collection

Plants were cultured in quarter‐strength Murashige and Skoog medium for 3 weeks. Leaf phloem exudates were collected from the second and third leaflets from the top of nine 5‐week‐old plants, and placed into a tube containing 0.5 mL of 10 mm ethylenediaminetetraacetic acid (EDTA) for 24 h (Urquhart and Joy, 1981). The exudates from three plants were pooled as one sample. Phloem exudation rates were determined as nanomoles of amino acid exudate per milligram of leaf tissue [dry weight (DW)] per hour. Root xylem exudates were collected from nine 4‐week‐old plants by cutting the hypocotyl below the rosette. To prevent contamination of the wound exudate, the first drop of fluid was discarded, after which 100‐μL fluid samples were collected during a 3‐h period with a 1‐mL needle tube. The exudates of each plant were then pooled and analysed (Abeysekara and Bhattacharyya, 2014).

### Transgenic soybean hairy root production

Transgenic soybean hairy roots were generated by soybean transformation using *Agrobacterium rhizogenes* strain K599 in accordance with previously described methods (Kereszt *et al*., 2007).

### GUS staining

Histochemical GUS assays were performed as described by Jefferson *et al*. (1987). The tissues were incubated for 8–12 h at 37 °C using a GUS staining kit (Huayueyang, China). An ethanol wash was performed overnight to clean tissue samples. The stained soybean hairy roots were subsequently embedded in 3% agarose and sectioned by hand.

### Confocal microscopy analysis

The construct P_35S_‐GmAAT‐GFP was used for transient expression in tobacco epidermal cells by injection via *Agrobacterium tumefaciens *GV3101 (Bai *et al*., 2014; Hernández‐Sánchez *et al*., 2017). For nuclear staining, DAPI reagent (Sigma, USA) was used. The tobacco epidermis was incubated in 5 μg/mL of DAPI for 5 min. GFP and DAPI were observed using a Nikon A1 spectral confocal microscope (Nikon, Japan) at an excitation wavelength of 488 nm and an emission wavelength of 509 nm for GFP, and at an excitation wavelength of 405 nm and an emission wavelength of 492 nm for DAPI.

### Quantitative analysis of JA

The soybean seedlings were cultured in quarter‐strength Murashige and Skoog medium for 4 weeks. The roots were harvested, rinsed briefly in phosphate‐buffered solution, immediately frozen in liquid nitrogen and ground into a powder, after which they were extracted with methanol–water (8 : 2) at 4 °C. Endogenous jasmonates (JA, MeJA and JA‐Ile) were quantified using HPLC‐MS/MS as described previously (Fang *et al*., 2016). Three biological replicates per line were analysed. Detection of JA was conducted by Metware Company (China).

### SCN assays

The SCN assays were performed in a glasshouse in accordance with well‐established methods (Arelli *et al*., 1997; Niblack *et al*., 2009). SCN HG type 0 (race 3) was used in this study. Briefly, infective second‐stage juveniles (J2s) were hatched from eggs and were used to inoculate soybean seedling with 2000 J2s per seedling. Thirty days after inoculation, female cysts were collected and quantified using a fluorescence‐based imaging system (Brown *et al*., 2010). Experiments were repeated at least twice.

### Chemical treatments

The treatment with nPG was designed on the basis of a previous report (Kinkema and Gresshoff, 2008). nPG, an inhibitor of JA biosynthesis, was dissolved in ethanol to make a 0.5 m stock solution. Prior to infection, 12‐day‐old soybean seedlings were immersed in quarter‐strength Murashige and Skoog medium and treated with either water containing 0.02% ethanol (control) or a 100 μm nPG solution daily for 3 days. These seedlings were then used to perform SCN assays.

## Author Contributions

W.G. and Y.J. designed the research. W.G., F.Z., Q.Y., Y.C., J.C., X.Z., X.S. and Y.J. performed the research. A.B. transformed the soybean plants. Z.L. and J.C. performed the nematode assays. W.Z. and W.G. analysed the data. W.G. and Y.J. wrote the paper. Y.J. coordinated the study.

## Supporting information


**Fig. S1** ****Amino acid alignment of the soybean Rhg1‐GmAAT (Glyma.18G022400) protein with AtAVT6C (At3G56200) and AtAVT6D (At2G40420) from *Arabidopsis*. ‘*’, identical residues; ‘:’, conserved substitution between similar residues; ‘.’, semi‐conserved substitutions between similar residues. The predicted amino acid transporter domain is highlighted in grey.

**Fig. S2** ****Subcellular localization of Rhg1‐GmAAT. Subcellular localization via a P_35S_‐GmAAT‐GFP fusion protein in onion epidermal cells (a) and soybean hairy roots (b). In (a), from left to right: 4,6‐diamidino‐2‐phenylindole (DAPI)‐stained nuclear DNA, green fluorescent protein (GFP) fluorescence, bright‐field and overlay panels. In (b), the fluorescence (left) and bright‐field (middle) images are overlaid on the right side (scale bar, 20 μm). Nucleus (N) and plasma membrane (P) are indicated by white and yellow arrows, respectively.

**Fig. S3** ****Expression of *Rhg1‐GmAAT*. (a) The expression of *Rhg1‐GmAAT* was determined by real‐time quantitative reverse transcription‐polymerase chain reaction (qRT‐PCR) in ecotype Colombia‐0 (Col‐0) and the transgenic Rhg1‐GmAAT‐OX (Rhg1‐GmAAT‐overexpressing) lines (at‐1 and at‐3). The level of *ATACT7* transcript served as a loading control. (b–d) Expression of *Rhg1‐GmAAT* determined by qRT‐PCR in the wild‐type (cultivar Tianlong 1) and transgenic Rhg1‐GmAAT‐OX lines (gm‐2 and gm‐3) (b), two soybean near‐isogenic lines (NILs; NIL‐S and NIL‐R) (c), and Williams 82 and PI88788 (d). The expression levels of all samples were normalized to those of *SKIP16*. The values are the means ± standard deviations (SDs) (*n* = 3). WT, wild‐type (cultivar Tianlong 1).

**Fig. S4** ****Growth of *Arabidopsis* seedlings subjected to excess amounts of amino acids. Seedlings were grown on half‐strength Murashige and Skoog medium supplemented with 2 mm tyrosine (Tyr) or lysine (Lys), 4 mm leucine (Leu) or methionine (Met), 6 mm valine (Val), 10 mm phenylalanine (Phe), isoleucine (Ile), serine (Ser), threonine (Thr) or tryptophan (Trp), 20 mm histidine (His), 25 mm cysteine (Cys) or glycine (Gly), 50 mm asparagine (Asn) or aspartic acid (Asp), and 100 mm glutamine (Gln), alanine (Ala), proline (Pro), arginine (Arg) or glutamic acid (Glu). As controls, Col‐0 plants were grown on half‐strength Murashige and Skoog medium only. Images were taken at 21 days after treatment. Scale bar, 1 cm.

**Fig. S5** ****Growth of soybean seedlings subjected to excess amounts of amino acids. Twenty‐one‐day‐old wild‐type (cultivar Tianlong 1) and transgenic Rhg1‐GmAAT‐OX (Rhg1‐GmAAT‐overexpressing) line gm‐3 were inoculated in quarter‐strength Murashige and Skoog medium containing 50 mm aspartic acid (Asp) and glutamic acid (Glu), 75 mm glutamine (Gln), and 25 mm glycine (Gly). As controls, the wild‐type (cultivar Tianlong 1) plants were grown on quarter‐strength Murashige and Skoog medium only. Images were taken at 3 days after treatment. Scale bar, 5 cm. WT, wild‐type; OX, Rhg1‐GmAAT‐OX line gm‐3.

**Fig. S** **6** ****Responses of a pair of near‐isogenic lines (NILs) to soybean cyst nematodes (SCNs). NIL‐S, NIL‐R and Hutcheson soybean seedlings were transplanted into sterilized sand, after which each plant was inoculated with 2000 J2 nematodes. Female cysts were quantified after 30 days. The experiments were repeated at least three times, each producing similar results. The values are the means ± standard deviations (SDs) (*n* = 6). Asterisks indicate a statistically significant difference of NIL‐R compared with Hutcheson. **P < 0.01 (multiple *t*‐test followed by the Holm–Sidak *post hoc* test).

**Fig. S** **7** ****Kyoto Encyclopedia of Genes and Genomes (KEGG) pathway enrichment scatter diagram of up‐regulated differentially expressed genes (DEGs). Only the 20 most enriched pathways are displayed in the diagram. The degree of KEGG pathway enrichment is represented by the rich factor, Q‐value and the number of unigenes enriched in a particular KEGG pathway. The rich factor is the ratio of differentially expressed unigenes enriched in a pathway to the total number of annotated unigenes in that pathway. The greater the rich factor, the greater the degree of enrichment. The Q‐value indicates the corrected *P* value and ranges from zero to unity; a Q‐value closer to zero indicates more enrichment.

**Fig. S8** ****Jasmonic acid (JA) content in soybean roots. (a) JA content in a bulk (Bulk‐S) of susceptible soybean varieties (Hutcheson, Magellan and Williams 82) and a bulk (Bulk‐R) of naturally resistant soybean varieties (PI437655, PI495017C, PI209332, PI438503A and PI467312). (b) JA content in the roots of susceptible soybean variety Hutcheson and resistant soybean variety PI88788. Seedlings were cultured in quarter‐strength Murashige and Skoog medium for 4 weeks, after which roots were harvested. Endogenous JA was quantified by following metabolic profiling procedures. The values are the means ± standard deviations (SDs) (*n* = 6). Asterisks indicate a statistically significant difference of resistant soybean varieties compared with susceptible soybean varieties. **P < 0.01 (multiple *t*‐test followed by the Holm–Sidak *post hoc* test). CPS, counts per second.

**Fig. S9** ****Expression of jasmonic acid (JA) biosynthesis genes expressed in n‐propyl gallate (nPG)‐treated PI88788 roots. Twelve‐day‐old PI88788 seedlings were inoculated with either water containing 0.02% ethanol (mock) or a 100 μm nPG solution for 3 days. The total RNA was extracted from the roots. The expression levels of the genes of interest were assayed by quantitative reverse transcription‐polymerase chain reaction (qRT‐PCR). The expression levels of all samples were normalized to *SKIP16*. The values are the means ± standard deviations (SDs) (*n* = 3). Asterisks indicate a statistically significant difference of nPG‐treated roots compared with mock‐treated roots. *0.01 < *P* < 0.05, **P < 0.01 (multiple *t*‐test followed by the Holm–Sidak *post hoc* test).

**Fig. S1** **0  **Expression of *Rhg1‐GmWI12* (*Glyma.18G022700*) induced by jasmonic acid (JA). Twelve‐day‐old Williams 82 soybean seedlings were cultured in quarter‐strength Murashige and Skoog medium that contained either 50 μm JA or water containing 0.02% ethanol (as a control). The roots were sampled after 8 h, after which their total RNA was extracted. The expression of the genes of interest was assayed by real‐time quantitative reverse transcription‐polymerase chain reaction (qRT‐PCR). The expression levels of all samples were normalized to *SKIP16*. The values are the means ± standard deviations (SDs) (*n* = 3). Asterisks indicate a statistically significant difference of JA‐treated roots compared with control roots. *0.01 < *P* < 0.05, **P < 0.01 (multiple *t*‐test followed by the Holm–Sidak *post hoc* test).

**Table S1** ****Free amino acid contents in the roots and leaves of 21‐day‐old wild‐type (cultivar Tianlong 1) and Rhg1‐GmAAT‐overexpressing (Rhg1‐GmAAT‐OX) line gm‐3. Amino acid contents were expressed as nanomoles per milligram. Root and leaf measurements were calculated using the dry weight (DW). Four plants constituted a sample, and three samples per line were used for the experiments. The values are the means ± standard deviations (SDs) (*n* = 3). The values shown in bold are significantly (*P* < 0.05) higher than those of the wild‐type (multiple *t*‐test followed by the Holm–Sidak *post hoc* test). This table contains the data presented in Fig. 4c,d, as well as additional information about the amino acids not presented in Fig. 4c,d. WT, wild‐type (cultivar Tianlong 1); OX, Rhg1‐GmAAT‐OX line gm‐3.

**Table S2** ****RNA‐sequencing (RNA‐Seq) results regarding changes in the expression of jasmonic acid (JA)‐related genes and *Rhg1* genes on *Rhg1‐GmAAT* overexpression. The values are the means ± standard deviations (SDs) (*n* = 3). FDR, false discovery rate.

**Table S3** ****Metabolite analysis results regarding changes in hormone contents on *Rhg1‐GmAAT* overexpression. The values are the means ± standard deviations (SDs) (*n* = 3). CPS, counts per second; VIP, variable importance in projection.

**Table S4** ****Oligonucleotide primers used in this study.

**Methods S1** Multiple alignments.

**Methods S2** DNA construct.

**Methods S3** RNA‐sequencing (RNA‐Seq): sample preparation and data analysis.

**Methods S4** Metabolic profiling.

## References

[mpp12753-bib-0001] Abeysekara, N.S. and Bhattacharyya, M.K. (2014) Analyses of the xylem sap proteomes identified candidate *Fusarium virguliforme *proteinaceous toxins. PLoS One, 9, e93667. 24845418 10.1371/journal.pone.0093667 PMC4028188

[mpp12753-bib-0002] Afzal, A.J. , Natarajan, A. , Saini, N. , Iqbal, M.J. , Geisler, M. , El Shemy, H.A. , Mungur, R. , Willmitzer, L. and Lightfoot, D.A. (2009) The nematode resistance allele at the *rhg1* locus alters the proteome and primary metabolism of soybean roots. Plant Physiol. 151, 1264–1280. 19429603 10.1104/pp.109.138149 PMC2773059

[mpp12753-bib-0003] Agut, B. , Gamir, J. , Jaques, J.A. and Flors, V. (2016) Systemic resistance in citrus to *Tetranychus urticae* induced by conspecifics is transmitted by grafting and mediated by mobile amino acids. J. Exp. Bot. 67, 5711–5723. 27683726 10.1093/jxb/erw335 PMC5066491

[mpp12753-bib-0004] Arelli, A.P. , Wilcox, J.A. , Myers, O. and Gibson, P.T. (1997) Soybean germplasm resistant to races 1 and 2 of *Heterodera glycines* . Crop Sci. 37, 1367–1369.

[mpp12753-bib-0005] Avanci, N.C. , Luche, D.D. , Goldman, G.H. and Goldman, M.H. (2010) Jasmonates are phytohormones with multiple functions, including plant defense and reproduction. Genet. Mol. Res. 9, 484–505. 20391333 10.4238/vol9-1gmr754

[mpp12753-bib-0006] Bai, L. , Ma, X. , Zhang, G. , Song, S. , Zhou, Y. , Gao, L. , Miao, Y. and Song, C.P. (2014) A receptor‐like kinase mediates ammonium homeostasis and is important for the polar growth of root hairs in *Arabidopsis* . Plant Cell, 26, 1497–1511. 24769480 10.1105/tpc.114.124586 PMC4036567

[mpp12753-bib-0007] Bayless, A.M. , Smith, J.M. , Song, J. , McMinn, P.H. , Teillet, A. , August, B.K. and Bent, A.F. (2016) Disease resistance through impairment of alpha‐SNAP‐NSF interaction and vesicular trafficking by soybean *Rhg1* . Proc. Natl. Acad. Sci. USA, 113, E7375–E7382. 27821740 10.1073/pnas.1610150113 PMC5127302

[mpp12753-bib-0008] Besnard, J. , Pratelli, R. , Zhao, C. , Sonawala, U. , Collakova, E. , Pilot, G. and Okumoto, S. (2016) UMAMIT14 is an amino acid exporter involved in phloem unloading in *Arabidopsis* roots. J. Exp. Bot. 67, 6385–6397. 27856708 10.1093/jxb/erw412 PMC5181585

[mpp12753-bib-0009] Brown, S. , Yeckel, G. , Heinz, R. , Clark, K. , Sleper, D. and Mitchum, M.G. (2010) A high‐throughput automated technique for counting females of *Heterodera glycines* using a fluorescence‐based imaging system. J. Nematol. 42, 201–206. 22736857 PMC3380484

[mpp12753-bib-0010] Brucker, E. , Niblack, T. , Kopisch‐Obuch, F.J. and Diers, B.W. (2005) The effect of rhg1 on reproduction of* Heterodera glycines* in the field and greenhouse and associated effects on agronomic traits. Crop Sci. 45, 1721–1727.

[mpp12753-bib-0011] Bruinsma, M. , van Loon, J.J.A. and Dicke, M. (2010) Increasing insight into induced plant defense mechanisms using elicitors and inhibitors. Plant Signal. Behav. 5, 271–274. 20081352 10.4161/psb.5.3.10623 PMC2881275

[mpp12753-bib-0012] Burstenbinder, K. , Rzewuski, G. , Wirtz, M. , Hell, R. and Sauter, M. (2007) The role of methionine recycling for ethylene synthesis in *Arabidopsis* . Plant J. 49, 238–249. 17144895 10.1111/j.1365-313X.2006.02942.x

[mpp12753-bib-0013] Caldwell, B.E. , Brim, C.A. and Ross, J.P. (1960) Inheritance of resistance of soybeans to the cyst nematode *Heterodera glycines* . Agron. J. 52, 635–636.

[mpp12753-bib-0014] Chahomchuen, T. , Hondo, K. , Ohsaki, M. , Sekito, T. and Kakinuma, Y. (2009) Evidence for Avt6 as a vacuolar exporter of acidic amino acids in *Saccharomyces cerevisiae* cells. J. Gen. Appl. Microbiol. 55, 409–417. 20118605 10.2323/jgam.55.409

[mpp12753-bib-0015] Chen, L. and Bush, D.R. (1997) LHT1, a lysine‐ and histidine‐specific amino acid transporter in *Arabidopsis* . Plant Physiol. 115, 1127–1134. 9390441 10.1104/pp.115.3.1127 PMC158577

[mpp12753-bib-0016] Chen, L. , Ortiz‐Lopez, A. , Jung, A. and Bush, D.R. (2001) ANT1, an aromatic and neutral amino acid transporter in *Arabidopsis* . Plant Physiol. 125, 1813–1820. 11299361 10.1104/pp.125.4.1813 PMC88837

[mpp12753-bib-0017] Chen, Q.J. , Zhou, H.M. , Chen, J. and Wang, X.C. (2006) Using a modified TA cloning method to create entry clones. Anal. Biochem. 358(1), 120–125. 16970900 10.1016/j.ab.2006.08.015

[mpp12753-bib-0018] Chen, S. , Songkumarn, P. , Liu, J. and Wang, G.L. (2009) A versatile zero background T‐vector system for gene cloning and functional genomics. Plant Physiol. 150, 1111–1121. 19403729 10.1104/pp.109.137125 PMC2705043

[mpp12753-bib-0021] Chen, Z. , Fei, Y.J. , Anderson, C.M.H. , Wake, K.A. , Miyauchi, S. , Huang, W. , Thwaites, D.T. and Ganapathy, V. (2003) Structure, function and immunolocalization of a proton‐coupled amino acid transporter (hPAT1) in the human intestinal cell line Caco‐2. J. Physiol. 546, 349–361. 12527723 10.1113/jphysiol.2002.026500 PMC2342508

[mpp12753-bib-0022] Clough, S.J. and Bent, A.F. (1998) Floral dip: a simplified method for *Agrobacterium*‐mediated transformation of *Arabidopsis thaliana* . Plant J. 16, 735–743. 10069079 10.1046/j.1365-313x.1998.00343.x

[mpp12753-bib-0023] Concibido, V.C. , Diers, B.W. and Arelli, P.R. (2004) A decade of QTL mapping for cyst nematode resistance in soybean. Crop Sci. 44, 1121–1131.

[mpp12753-bib-0024] Cook, D.E. , Lee, T.G. , Guo, X. , Melito, S. , Wang, K. , Bayless, A.M. , Wang, J. , Hughes, T.J. , Willis, D.K. , Clemente, T.E. , Diers, B.W. , Jiang, J. , Hudson, M.E. and Bent, A.F. (2012) Copy number variation of multiple genes at *Rhg1* mediates nematode resistance in soybean. Science, 338, 1206–1209. 23065905 10.1126/science.1228746

[mpp12753-bib-0025] Cook, R. (2004) Genetic resistance to nematodes: where is it useful? Australas. Plant Pathol. 33, 139–150.

[mpp12753-bib-0026] Dundar, E. and Bush, D.R. (2009) BAT1, a bidirectional amino acid transporter in *Arabidopsis* . Planta, 229, 1047–1056. 19199104 10.1007/s00425-009-0892-8

[mpp12753-bib-0027] Fang, L. , Su, L. , Sun, X. , Li, X. , Sun, M. , Karungo, S.K. , Fang, S. , Chu, J. , Li, S. and Xin, H. (2016) Expression of *Vitis amurensis* NAC26 in *Arabidopsis* enhances drought tolerance by modulating jasmonic acid synthesis. J. Exp. Bot. 67, 2829–2845. 27162276 10.1093/jxb/erw122 PMC4861026

[mpp12753-bib-0028] Frommer, W.B. , Hummel, S. and Riesmeier, J.W. (1993) Expression cloning in yeast of a cDNA encoding a broad specificity amino acid permease from *Arabidopsis thaliana* . Proc. Natl. Acad. Sci. USA, 90, 5944–5948. 8327465 10.1073/pnas.90.13.5944 PMC46843

[mpp12753-bib-0029] Frommer, W.B. , Hummel, S. , Unseld, M. and Ninnemann, O. (1995) Seed and vascular expression of a high‐affinity transporter for cationic amino acids in *Arabidopsis* . Proc. Natl. Acad. Sci. USA, 92, 12 036–12 040. 10.1073/pnas.92.26.12036 PMC40291 8618839

[mpp12753-bib-0030] Gfeller, A. , Dubugnon, L. , Liechti, R. and Farmer, E.E. (2010) Jasmonate biochemical pathway. Sci. Signal. 3, cm3. 20159849 10.1126/scisignal.3109cm3

[mpp12753-bib-0031] Gheysen, G. and Mitchum, M.G. (2011) How nematodes manipulate plant development pathways for infection. Curr. Opin. Plant Biol. 14, 415–421. 21458361 10.1016/j.pbi.2011.03.012

[mpp12753-bib-0032] Gleason, C. , Leelarasamee, N. , Meldau, D. and Feussner, I. (2016) OPDA has key role in regulating plant susceptibility to the root‐knot nematode *Meloidogyne hapla* in *Arabidopsis* . Front. Plant Sci. 7, 1565. 27822219 10.3389/fpls.2016.01565 PMC5075541

[mpp12753-bib-0034] Heil, M. and Ton, J. (2008) Long‐distance signalling in plant defence. Trends Plant Sci. 13, 264–272. 18487073 10.1016/j.tplants.2008.03.005

[mpp12753-bib-0035] Hernández‐Sánchez, L.E. , Maruri‐López, I. , Graether, S.P. and Jiménez‐Bremont, J.F. (2017) In vivo evidence for homo‐ and heterodimeric interactions of *Arabidopsis thaliana* dehydrins AtCOR47, AtERD10, and AtRAB18. Sci. Rep. 7, 1–13. 29213048 10.1038/s41598-017-15986-2 PMC5719087

[mpp12753-bib-0036] Hirner, A. , Ladwig, F. , Stransky, H. , Okumoto, S. , Keinath, M. , Harms, A. , Frommer, W.B. and Koch, W. (2006) *Arabidopsis* LHT1 is a high‐affinity transporter for cellular amino acid uptake in both root epidermis and leaf mesophyll. Plant Cell, 18, 1931–1946. 16816136 10.1105/tpc.106.041012 PMC1533986

[mpp12753-bib-0037] Ithal, N. , Recknor, J. , Nettleton, D. , Hearne, L. , Maier, T. , Baum, T.J. and Mitchum, M.G. (2007a) Parallel genome‐wide expression profiling of host and pathogen during soybean cyst nematode infection of soybean. Mol. Plant–Microbe Interact. 20, 293–305. 17378432 10.1094/MPMI-20-3-0293

[mpp12753-bib-0038] Ithal, N. , Recknor, J. , Nettleton, D. , Maier, T. , Baum, T.J. and Mitchum, M.G. (2007b) Developmental transcript profiling of cyst nematode feeding cells in soybean roots. Mol. Plant–Microbe Interact. 20, 510–525. 17506329 10.1094/MPMI-20-5-0510

[mpp12753-bib-0039] Jefferson, R.A. , Kavanagh, T.A. and Bevan, M.W. (1987) GUS fusions: beta‐glucuronidase as a sensitive and versatile gene fusion marker in higher plants. EMBO J. 6, 3901–3907. 3327686 10.1002/j.1460-2075.1987.tb02730.x PMC553867

[mpp12753-bib-0040] Jensen, A. , Figueiredo‐Larsen, M. , Holm, R. , Broberg, M.L. , Brodin, B. and Nielsen, C.U. (2014) PAT1 (SLC36A1) shows nuclear localization and affects growth of smooth muscle cells from rats. Am. J. Physiol. Renal Physiol. 306, E65–E74. 10.1152/ajpendo.00322.2013 24222668

[mpp12753-bib-0041] Kadotani, N. , Akagi, A. , Takatsuji, H. , Miwa, T. and Igarashi, D. (2016) Exogenous proteinogenic amino acids induce systemic resistance in rice. BMC Plant Biol. 16, 60. 26940322 10.1186/s12870-016-0748-x PMC4778346

[mpp12753-bib-0042] Kammerhofer, N. , Radakovic, Z. , Regis, J.M. , Dobrev, P. , Vankova, R. , Grundler, F.M. , Siddique, S. , Hofmann, J. and Wieczorek, K. (2015) Role of stress‐related hormones in plant defence during early infection of the cyst nematode *Heterodera schachtii* in *Arabidopsis* . New Phytol. 207, 778–789. 25825039 10.1111/nph.13395 PMC4657489

[mpp12753-bib-0043] Karimi, M. , Inze, D. and Depicker, A. (2002) GATEWAY vectors for *Agrobacterium*‐mediated plant transformation. Trends Plant Sci. 7, 193–195. 11992820 10.1016/s1360-1385(02)02251-3

[mpp12753-bib-0044] Kereszt, A. , Li, D. , Indrasumunar, A. , Nguyen, C.D. , Nontachaiyapoom, S. , Kinkema, M. and Gresshoff, P.M. (2007) *Agrobacterium rhizogenes*‐mediated transformation of soybean to study root biology. Nat. Protoc. 2, 948–952. 17446894 10.1038/nprot.2007.141

[mpp12753-bib-0045] Kim, M. , Hyten, D.L. , Bent, A.F. and Diers, B.W. (2010) Fine mapping of the SCN resistance locus rhg1‐b from PI88788. Plant Genome, 3, 81–89.

[mpp12753-bib-0046] Kinkema, M. and Gresshoff, P.M. (2008) Investigation of downstream signals of the soybean autoregulation of nodulation receptor kinase GmNARK. Mol. Plant–Microbe Interact. 21, 1337–1348. 18785829 10.1094/MPMI-21-10-1337

[mpp12753-bib-0047] Klink, V.P. , Hosseini, P. , Matsye, P. , Alkharouf, N.W. and Matthews, B.F. (2009) A gene expression analysis of syncytia laser microdissected from the roots of the *Glycine max *(soybean) genotype PI 548402 (Peking) undergoing a resistant reaction after infection by* Heterodera glycines *(soybean cyst nematode). Plant Mol. Biol. 71, 525–567. 19787434 10.1007/s11103-009-9539-1

[mpp12753-bib-0048] Klink, V.P. , Hosseini, P. , Matsye, P.D. , Alkharouf, N.W. and Matthews, B.F. (2010) Syncytium gene expression in *Glycine max *(PI88788) roots undergoing a resistant reaction to the parasitic nematode* Heterodera glycines* . Plant Physiol. Biochem. 48, 176–193. 20138530 10.1016/j.plaphy.2009.12.003

[mpp12753-bib-0049] Kongrit, D. , Jisaka, M. , Iwanaga, C. , Yokomichi, H. , Katsube, T. , Nishimura, K. , Nagaya, T. and Yokota, K. (2007) Molecular cloning and functional expression of soybean allene oxide synthases. Biosci. Biotechnol. Biochem. 71, 491–498. 17284840 10.1271/bbb.60537

[mpp12753-bib-0050] Ladwig, F. , Stahl, M. , Ludewig, U. , Hirner, A.A. , Hammes, U.Z. , Stadler, R. , Harter, K. and Koch, W. (2012) Siliques are Red1 from *Arabidopsis* acts as a bidirectional amino acid transporter that is crucial for the amino acid homeostasis of siliques. Plant Physiol. 158, 1643–1655. 22312005 10.1104/pp.111.192583 PMC3320175

[mpp12753-bib-0051] Lee, T.G. , Kumar, I. , Diers, B.W. and Hudson, M.E. (2015) Evolution and selection of *Rhg1*, a copy‐number variant nematode‐resistance locus. Mol. Ecol. 24, 1774–1791. 25735447 10.1111/mec.13138 PMC4413360

[mpp12753-bib-0052] Lee, Y.H. , Foster, J. , Chen, J. , Voll, L.M. , Weber, A.P. and Tegeder, M. (2007) AAP1 transports uncharged amino acids into roots of *Arabidopsis* . Plant J. 50, 305–319. 17419840 10.1111/j.1365-313X.2007.03045.x

[mpp12753-bib-0053] Lee, Y.H. and Tegeder, M. (2004) Selective expression of a novel high‐affinity transport system for acidic and neutral amino acids in the tapetum cells of *Arabidopsis* flowers. Plant J. 40, 60–74. 15361141 10.1111/j.1365-313X.2004.02186.x

[mpp12753-bib-0054] Less, H. and Galili, G. (2008) Principal transcriptional programs regulating plant amino acid metabolism in response to abiotic stresses. Plant Physiol. 147, 316–330. 18375600 10.1104/pp.108.115733 PMC2330312

[mpp12753-bib-0056] Li, S. , Chen, Y. , Zhu, X. , Wang, Y. , Jung, K.H. , Chen, L. , Xuan, Y. and Duan, Y. (2018) The transcriptomic changes of Huipizhi Heidou (*Glycine max*), a nematode‐resistant black soybean during* Heterodera glycines *race 3 infection. J. Plant Physiol. 220, 96–104. 29169106 10.1016/j.jplph.2017.11.001

[mpp12753-bib-0057] Lin, J. , Mazarei, M. , Zhao, N. , Zhu, J.J. , Zhuang, X. , Liu, W. , Pantalone, V.R. , Arelli, P.R. , Stewart, C.N. and Chen, F. (2013) Overexpression of a soybean salicylic acid methyltransferase gene confers resistance to soybean cyst nematode. Plant. Biotechnol. J. 11, 1135–1145. 24034273 10.1111/pbi.12108

[mpp12753-bib-0058] Liu, G. , Ji, Y. , Bhuiyan, N.H. , Pilot, G. , Selvaraj, G. , Zou, J. and Wei, Y. (2010) Amino acid homeostasis modulates salicylic acid‐associated redox status and defense responses in *Arabidopsis* . Plant Cell, 22, 3845–3863. 21097712 10.1105/tpc.110.079392 PMC3015111

[mpp12753-bib-0059] Liu, S. , Kandoth, P.K. , Lakhssassi, N. , Kang, J. , Colantonio, V. , Heinz, R. , Yeckel, G. , Zhou, Z. , Bekal, S. , Dapprich, J. , Rotter, B. , Cianzio, S. , Mitchum, M.G. and Meksem, K. (2017) The soybean *GmSNAP18* gene underlies two types of resistance to soybean cyst nematode. Nat. Commun. 8, 14 822. 28345654 10.1038/ncomms14822 PMC5378975

[mpp12753-bib-0060] Masclaux‐Daubresse, C. (2006) Glutamine synthetase‐glutamate synthase pathway and glutamate dehydrogenase play distinct roles in the sink–source nitrogen cycle in tobacco. Plant Physiol. 140, 444–456. 16407450 10.1104/pp.105.071910 PMC1361315

[mpp12753-bib-0061] Matthews, B.F. , Beard, H. , Brewer, E. , Kabir, S. , MacDonald, M.H. and Youssef, R.M. (2014) Arabidopsis genes, *AtNPR1, AtTGA2 and AtPR‐5*, confer partial resistance to soybean cyst nematode (*Heterodera glycines*) when overexpressed in transgenic soybean roots. BMC Plant Biol. 14, 96. 24739302 10.1186/1471-2229-14-96 PMC4021311

[mpp12753-bib-0062] Meksem, K. , Pantazopoulos, P. , Njiti, V.N. , Hyten, L.D. , Arelli, P.R. and Lightfoot, D.A. (2001) ’Forrest’ resistance to the soybean cyst nematode is bigenic: saturation mapping of the *Rhg1 *and *Rhg4 *loci. Theor. Appl. Genet. 103, 710–717.

[mpp12753-bib-0063] Michaeli, S. , Fait, A. , Lagor, K. , Nunes‐Nesi, A. , Grillich, N. , Yellin, A. , Bar, D. , Khan, M. , Fernie, A.R. , Turano, F.J. and Fromm , H. (2011) A mitochondrial GABA permease connects the GABA shunt and the TCA cycle, and is essential for normal carbon metabolism. Plant J. 67, 485–498. 21501262 10.1111/j.1365-313X.2011.04612.x

[mpp12753-bib-0064] Nahar, K. , Kyndt, T. , De Vleesschauwer, D. , Hofte, M. and Gheysen, G. (2011) The jasmonate pathway is a key player in systemically induced defense against root knot nematodes in rice. Plant Physiol. 157, 305–316. 21715672 10.1104/pp.111.177576 PMC3165880

[mpp12753-bib-0065] Niblack, T.L. , Lambert, K.N. and Tylka, G.L. (2006) A model plant pathogen from the kingdom Animalia:* Heterodera glycines*, the soybean cyst nematode. Annu. Rev. Phytopathol. 44, 283–303. 16704359 10.1146/annurev.phyto.43.040204.140218

[mpp12753-bib-0066] Niblack, T. , Tylka, G.L. , Arelli, P. , Bond, J. , Diers, B. , Donald, P. , Faghihi, J. , Ferris, V.R. , Gallo, K. , Heinz, R.D. , Lopez‐Nicora, H. , VonQualen, R. , Welacky, T. and Wilcox, J. (2009) A standard greenhouse method for assessing soybean cyst nematode resistance in soybean: SCE08 (Standardized Cyst Evaluation 2008). Plant Health Prog. 10(1), 33.

[mpp12753-bib-0067] Okumoto, S. and Pilot, G. (2011) Amino acid export in plants: a missing link in nitrogen cycling. Mol. Plant, 4, 453–463. 21324969 10.1093/mp/ssr003 PMC3143828

[mpp12753-bib-0068] Okumoto, S. , Schmidt, R. , Tegeder, M. , Fischer, W.N. , Rentsch, D. , Frommer, W.B. and Koch, W. (2002) High affinity amino acid transporters specifically expressed in xylem parenchyma and developing seeds of *Arabidopsis* . J. Biol. Chem. 277, 45 338–45 346. 10.1074/jbc.M207730200 12244056

[mpp12753-bib-0069] Ortiz‐Lopez, A. , Chang, H. and Bush, D.R. (2002) Amino acid transporters in plants. Biochim. Biophys. Acta, 1465, 275–280. 10.1016/s0005-2736(00)00144-9 10748260

[mpp12753-bib-0070] Pauwels, L. and Goossens, A. (2011) The JAZ proteins: a crucial interface in the jasmonate signaling cascade. Plant Cell, 23, 3089. 21963667 10.1105/tpc.111.089300 PMC3203442

[mpp12753-bib-0071] Paz, M.M. , Martinez, J.C. , Kalvig, A.B. , Fonger, T.M. and Wang, K. (2006) Improved cotyledonary node method using an alternative explant derived from mature seed for efficient *Agrobacterium*‐mediated soybean transformation. Plant Cell Rep. 25, 206–213. 16249869 10.1007/s00299-005-0048-7

[mpp12753-bib-0072] Pena‐Cortés, H. , Albrecht, T. , Prat, S. , Weiler, E.W. and Willmitzer, L. (1993) Aspirin prevents wound‐induced gene expression in tomato leaves by blocking jasmonic acid biosynthesis. Planta, 191, 123–128.

[mpp12753-bib-0074] Perchlik, M. , Foster, J. and Tegeder, M. (2014) Different and overlapping functions of *Arabidopsis* LHT6 and AAP1 transporters in root amino acid uptake. J. Exp. Bot. 65, 5193–5204. 25005136 10.1093/jxb/eru278 PMC4157705

[mpp12753-bib-0076] Puthoff, D.P. , Nettleton, D. , Rodermel, S.R. and Baum, T.J. (2003) *Arabidopsis* gene expression changes during cyst nematode parasitism revealed by statistical analyses of microarray expression profiles. Plant J. 33, 911–921. 12609032 10.1046/j.1365-313x.2003.01677.x

[mpp12753-bib-0077] Rawat, S.R. , Silim, S.N. , Kronzucker, H.J. , Siddiqi, M.Y. and Glass, A.D. (1999) *AtAMT1* gene expression and NH_4_ ^+^ uptake in roots of *Arabidopsis thaliana*: evidence for regulation by root glutamine levels. Plant J. 19, 143–152. 10476061 10.1046/j.1365-313x.1999.00505.x

[mpp12753-bib-0078] Sauer, M. , Robert, S. and Kleine‐Vehn, J. (2013) Auxin: simply complicated. J. Exp. Bot. 64, 2565–2577. 23669571 10.1093/jxb/ert139

[mpp12753-bib-0079] Schilmiller, A.L. and Howe, G.A. (2005) Systemic signaling in the wound response. Curr. Opin. Plant Biol. 8, 369–377. 15939667 10.1016/j.pbi.2005.05.008

[mpp12753-bib-0080] Schmidt, R. , Stransky, H. and Koch, W. (2007) The amino acid permease AAP8 is important for early seed development in *Arabidopsis thaliana* . Planta, 226, 805–813. 17476526 10.1007/s00425-007-0527-x

[mpp12753-bib-0081] Seifi, H.S. , Van Bockhaven, J. , Angenon, G. and Hofte, M. (2013) Glutamate metabolism in plant disease and defense: friend or foe? Mol. Plant–Microbe Interact. 26, 475–485. 23342972 10.1094/MPMI-07-12-0176-CR

[mpp12753-bib-0082] Sidonskaya, E. , Schweighofer, A. , Shubchynskyy, V. , Kammerhofer, N. , Hofmann, J. , Wieczorek, K. and Meskiene, I. (2016) Plant resistance against the parasitic nematode* Heterodera schachtii* is mediated by MPK3 and MPK6 kinases, which are controlled by the MAPK phosphatase AP2C1 in *Arabidopsis* . J. Exp. Bot. 67, 107–118. 26438412 10.1093/jxb/erv440 PMC4682428

[mpp12753-bib-0083] Simpson, R.J. (1986) Translocation and metabolism of nitrogen: whole plant aspects In: Fundamental, Ecological and Agricultural Aspects of Nitrogen Metabolism in Higher Plants (Lambers H., Neeteson J.J. and Stulen I., eds), pp. 71–96. Dordrecht: Springer Netherlands.

[mpp12753-bib-0084] Song, H. , Wang, P. , Li, C. , Han, S. , Lopez‐Baltazar, J. , Zhang, X. and Wang, X. (2016) Identification of lipoxygenase (*LOX*) genes from legumes and their responses in wild type and cultivated peanut upon *Aspergillus flavus* infection. Sci. Rep. 6, 35 245. 10.1038/srep35245 PMC5059700 27731413

[mpp12753-bib-0086] Spitzner, A. , Perzlmaier, A.F. , Geillinger, K.E. , Reihl, P. and Stolz, J. (2008) The proline‐dependent transcription factor Put3 regulates the expression of the riboflavin transporter MCH5 in *Saccharomyces cerevisiae* . Genetics, 180, 2007–2017. 18940788 10.1534/genetics.108.094458 PMC2600938

[mpp12753-bib-0087] Su, Y.H. , Frommer, W.B. and Ludewig, U. (2004) Molecular and functional characterization of a family of amino acid transporters from *Arabidopsis* . Plant Physiol. 136, 3104–3113. 15377779 10.1104/pp.104.045278 PMC523371

[mpp12753-bib-0088] Svennerstam, H. , Ganeteg, U. and Nasholm, T. (2008) Root uptake of cationic amino acids by *Arabidopsis* depends on functional expression of amino acid permease 5. New Phytol. 180, 620–630. 18681934 10.1111/j.1469-8137.2008.02589.x

[mpp12753-bib-0089] Svennerstam, H. , Jamtgard, S. , Ahmad, I. , Huss‐Danell, K. , Nasholm, T. and Ganeteg, U. (2011) Transporters in *Arabidopsis* roots mediating uptake of amino acids at naturally occurring concentrations. New Phytol. 191, 459–467. 21453345 10.1111/j.1469-8137.2011.03699.x

[mpp12753-bib-0090] Taki, N. , Sasaki‐Sekimoto, Y. , Obayashi, T. , Kikuta, A. , Kobayashi, K. , Ainai, T. , Yagi, K. , Sakurai, N. , Suzuki, H. , Masuda, T. and Takamiya , K.I. (2005) 12‐oxo‐phytodienoic acid triggers expression of a distinct set of genes and plays a role in wound‐induced gene expression in *Arabidopsis* . Plant Physiol. 139, 1268–1283. 16258017 10.1104/pp.105.067058 PMC1283764

[mpp12753-bib-0092] Tegeder, M. , Ruan, Y.L. and Patrick, J.W. (2012) Roles of plasma membrane transporters in phloem functions In: Phloem: Molecular Cell Biology, Systemic Communication, Biotic Interactions (Thompson G. and van Bel A.J.E., eds), pp. 61–101. Oxford: Wiley‐Blackwell.

[mpp12753-bib-0093] Tylka, G.L. , Gebhart, G.D. , Marett, C.C. , Mullaney, M.P. and Wiggs, S.N. (2010) Evaluation of Soybean Varieties Resistant to Soybean Cyst Nematode in Iowa‐2010, 52 Ames, IA: Iowa State University Extension and Outreach IPM .

[mpp12753-bib-0094] Urquhart, A.A. and Joy, K.W. (1981) Use of phloem exudate technique in the study of amino acid transport in pea plants. Plant Physiol. 68, 750–754. 16661993 10.1104/pp.68.3.750 PMC425975

[mpp12753-bib-0095] Verhage, A. , Vlaardingerbroek, I. , Raaymakers, C. , Van Dam, N.M. , Dicke, M. , Van Wees, S.C. and Pieterse, C.M. (2011) Rewiring of the jasmonate signaling pathway in *Arabidopsis* during insect herbivory. Front. Plant Sci. 2, 47. 22645537 10.3389/fpls.2011.00047 PMC3355780

[mpp12753-bib-0096] Wasternack, C. and Hause, B. (2013) Jasmonates: biosynthesis, perception, signal transduction and action in plant stress response, growth and development. An update to the 2007 review in Ann. Bot.. Ann. Bot. 111, 1021–1058. 23558912 10.1093/aob/mct067 PMC3662512

[mpp12753-bib-0097] Webb, D.M. , Baltazar, B.M. , Rao‐Arelli, A.P. , Schupp, J. , Clayton, K. , Keim, P. and Beavis, W.D. (1995) Genetic mapping of soybean cyst nematode race‐3 resistance loci in the soybean PI 437.654. Theor. Appl. Genet. 91, 574–581. 24169883 10.1007/BF00223282

[mpp12753-bib-0098] Yen, S. , Chung, M. , Chen, P. and Yen, H.E. (2001) Environmental and developmental regulation of the wound‐induced cell wall protein WI12 in the halophyte ice plant. Plant Physiol. 127, 517–528. 11598226 PMC125087

[mpp12753-bib-0099] Zhang, H. , Kjemtrup‐Lovelace, S. , Li, C. , Luo, Y. , Chen, L.P. and Song, B.H. (2017) Comparative RNA‐Seq analysis uncovers a complex regulatory network for soybean cyst nematode resistance in wild soybean (*Glycine soja*). Sci. Rep. 7, 9699. 28852059 10.1038/s41598-017-09945-0 PMC5575055

