# Supplementary material for: The soybean Rhg1 amino acid transporter gene alters glutamate homeostasis and jasmonic acid‐induced resistance to soybean cyst nematode
Source: Mol Plant Pathol. 2018 Nov 15;20(2):270–86. doi: 10.1111/mpp.12753 (PMC6637870; doi:10.1111/mpp.12753)
Supplement: Supplementary file 1 — Fig. S1 Amino acid alignment of the soybean Rhg1‐GmAAT (Glyma.18G022400) protein with AtAVT6C (At3G56200) and AtAVT6D (At2G40420) from Arabidopsis. ‘*’, identical residues; ‘:’, conserved substitution between similar residues; ‘.’, semi‐conserved substitutions between similar residues. The predicted amino acid transporter domain is highlighted in grey. [file MPP-20-270-s001.docx]

**
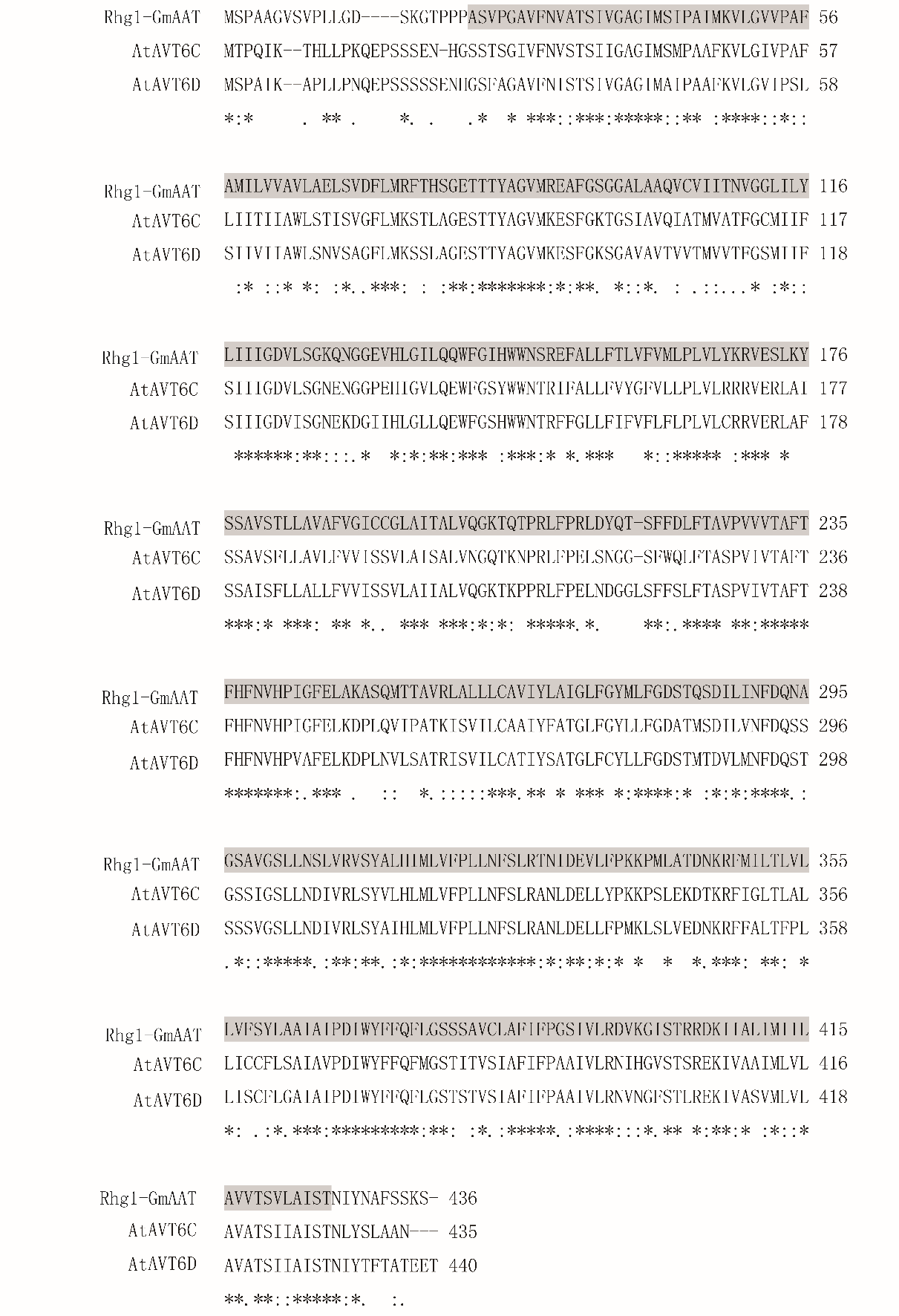
**

**Figure S1. Amino acid alignment of the soybean Rhg1-GmAAT (Glyma.18G022400) protein with AtAVT6C (At3G56200) and AtAVT6D (At2G40420) from *Arabidopsis*.** “*” represents identical residues; “:”means conserved substitution between similar residues; “.” indicates the semi-conserved substitutions between similar residues. The predicted amino acid transporter domain is highlighted in gray.
