# Supplementary material for: The soybean Rhg1 amino acid transporter gene alters glutamate homeostasis and jasmonic acid‐induced resistance to soybean cyst nematode
Source: Mol Plant Pathol. 2018 Nov 15;20(2):270–86. doi: 10.1111/mpp.12753 (PMC6637870; doi:10.1111/mpp.12753)
Supplement: Supplementary file 2 — Fig. S2 Subcellular localization of Rhg1‐GmAAT. Subcellular localization via a P35S‐GmAAT‐GFP fusion protein in onion epidermal cells (a) and soybean hairy roots (b). In (a), from left to right: 4,6‐diamidino‐2‐phenylindole (DAPI)‐stained nuclear DNA, green fluorescent protein (GFP) fluorescence, bright‐field and overlay panels. In (b), the fluorescence (left) and bright‐field (middle) images are overlaid on the right side (scale bar, 20 μm). Nucleus (N) and plasma membrane (P) are indicated by white and yellow arrows, respectively. [file MPP-20-270-s002.docx]

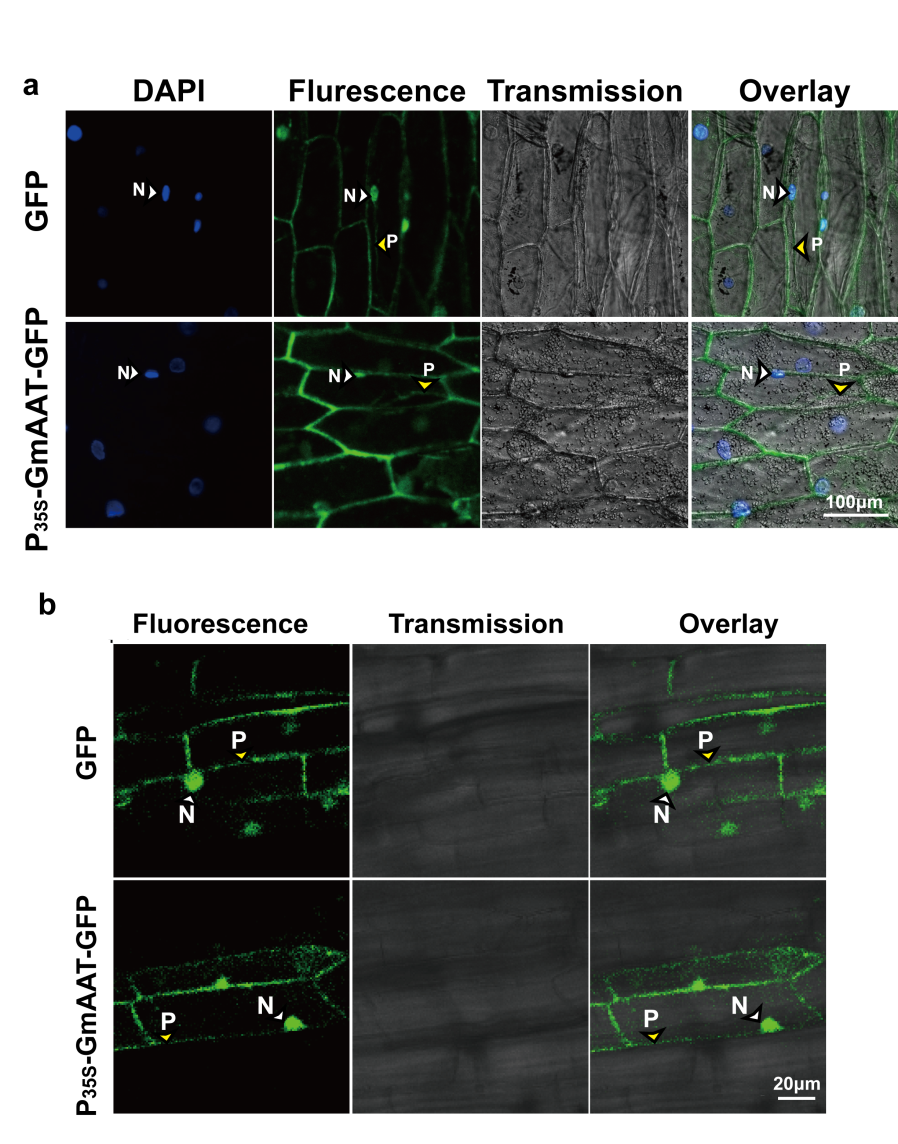


**Figure S2. Subcellular localization of Rhg1-GmAAT.** Subcellular localization via a P_35S_-GmAAT-GFP fusion protein in onion epidermal cells (a) and soybean hairy roots (b). In (a), from left to right: DAPI (4, 6-diamidino-2-phenylindole)-stained nuclear DNA, GFP fluorescence, bright-field and overlay panels. In (b), the fluorescence (left) and bright-field (middle) images are overlaid on the right side (scale bar=20 μm). Nucleus (N), and plasma membrane (P) were indicated by white and yellow arrows respectively.
