# Supplementary material for: The soybean Rhg1 amino acid transporter gene alters glutamate homeostasis and jasmonic acid‐induced resistance to soybean cyst nematode
Source: Mol Plant Pathol. 2018 Nov 15;20(2):270–86. doi: 10.1111/mpp.12753 (PMC6637870; doi:10.1111/mpp.12753)
Supplement: Supplementary file 3 — Fig. S3 Expression of Rhg1‐GmAAT. (a) The expression of Rhg1‐GmAAT was determined by real‐time quantitative reverse transcription‐polymerase chain reaction (qRT‐PCR) in ecotype Colombia‐0 (Col‐0) and the transgenic Rhg1‐GmAAT‐OX (Rhg1‐GmAAT‐overexpressing) lines (at‐1 and at‐3). The level of ATACT7 transcript served as a loading control. (b–d) Expression of Rhg1‐GmAAT determined by qRT‐PCR in the wild‐type (cultivar Tianlong 1) and transgenic Rhg1‐GmAAT‐OX lines (gm‐2 and gm‐3) (b), two soybean near‐isogenic lines (NILs; NIL‐S and NIL‐R) (c), and Williams 82 and PI88788 (d). The expression levels of all samples were normalized to those of SKIP16. The values are the means ± standard deviations (SDs) (n = 3). WT, wild‐type (cultivar Tianlong 1). [file MPP-20-270-s003.docx]

**
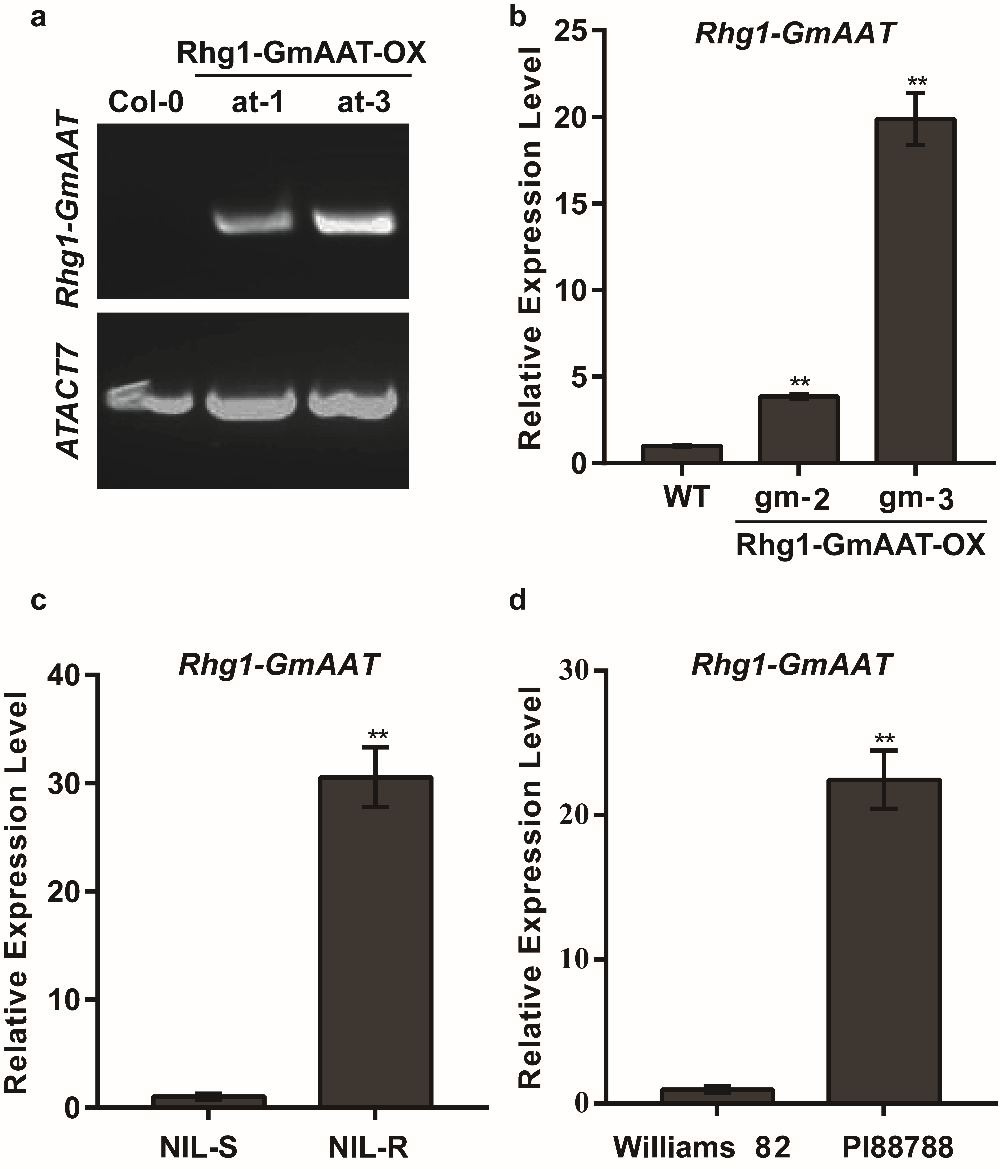
**

**Figure S3. Expression of *Rhg1-GmAAT*.** (a) The expression of *Rhg1-GmAAT* was determined by quantitative real-time PCR (RT-PCR) in ecotype Colombia-0 (Col-0) and the transgenic overexpressed Rhg1-GmAAT-OX lines (at-1 and at-3). The level of *ATACT7* transcript served as a loading control. (b) and (c) Expression of *Rhg1-GmAAT* determined by quantitative RT-PCR in the wild-type (cultivar Tianlong 1) and transgenic overexpressed Rhg1-GmAAT-OX lines (gm-2 and gm-3) (b), two soybean near isogenic lines (NILs; NIL-S and NIL-R) (c), and Williams 82 and PI88788 (d). The expression levels of all samples were normalized to those of *SKIP16*. The values were the means±SDs (n=3). WT, wild-type (cultivar Tianlong 1).
