# Supplementary material for: The soybean Rhg1 amino acid transporter gene alters glutamate homeostasis and jasmonic acid‐induced resistance to soybean cyst nematode
Source: Mol Plant Pathol. 2018 Nov 15;20(2):270–86. doi: 10.1111/mpp.12753 (PMC6637870; doi:10.1111/mpp.12753)
Supplement: Supplementary file 4 — Fig. S4 Growth of Arabidopsis seedlings subjected to excess amounts of amino acids. Seedlings were grown on half‐strength Murashige and Skoog medium supplemented with 2 mm tyrosine (Tyr) or lysine (Lys), 4 mm leucine (Leu) or methionine (Met), 6 mm valine (Val), 10 mm phenylalanine (Phe), isoleucine (Ile), serine (Ser), threonine (Thr) or tryptophan (Trp), 20 mm histidine (His), 25 mm cysteine (Cys) or glycine (Gly), 50 mm asparagine (Asn) or aspartic acid (Asp), and 100 mm glutamine (Gln), alanine (Ala), proline (Pro), arginine (Arg) or glutamic acid (Glu). As controls, Col‐0 plants were grown on half‐strength Murashige and Skoog medium only. Images were taken at 21 days after treatment. Scale bar, 1 cm. [file MPP-20-270-s004.docx]

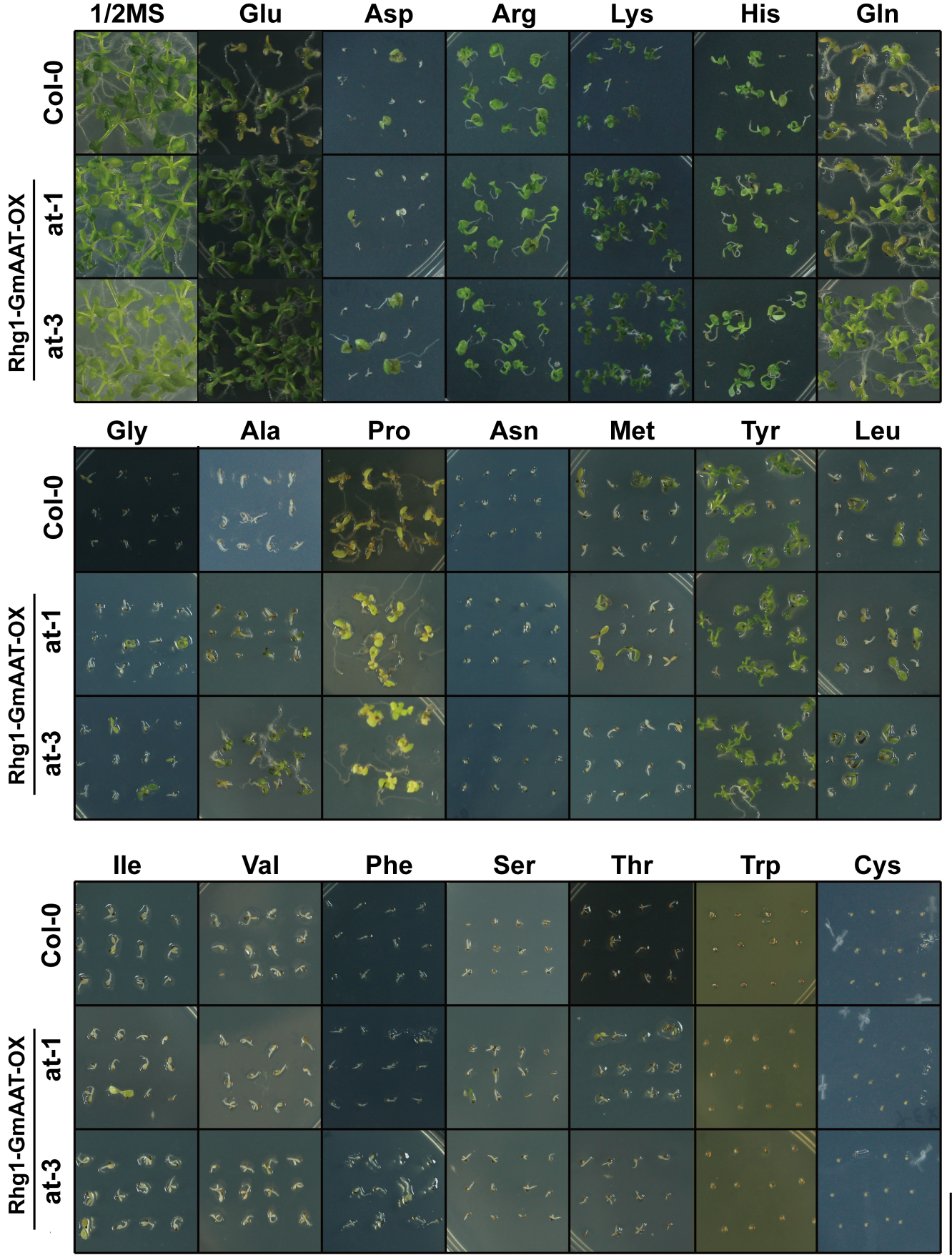


**Figure S4. Growth of *Arabidopsis* seedlings subjected to excess amounts of amino acids.** Seedlings were grown on 1/2 Murashige and Skoog (MS) medium that was supplemented with 2 mM tyrosine (Tyr) or lysine (Lys); 4 mM leucine (Leu) or methionine (Met); 6 mM valine (Val); 10 mM phenylalanine (Phe), isoleucine (Ile), serine (Ser), threonine (Thr) or tryptophan (Trp); 20 mM histidine (His); 25 mM cysteine (Cys) or glycine (Gly); 50 mM asparagine (Asn) or aspartate (Asp); and 100 mM glutamine (Gln), alanine (Ala), proline (Pro), arginine (Arg) or glutamate (Glu). As controls, Col-0 plants were grown on 1/2 MS medium only. Images were taken at 21 days after treatment. Scale bar=1 cm.
