# Supplementary material for: The soybean Rhg1 amino acid transporter gene alters glutamate homeostasis and jasmonic acid‐induced resistance to soybean cyst nematode
Source: Mol Plant Pathol. 2018 Nov 15;20(2):270–86. doi: 10.1111/mpp.12753 (PMC6637870; doi:10.1111/mpp.12753)
Supplement: Supplementary file 5 — Fig. S5 Growth of soybean seedlings subjected to excess amounts of amino acids. Twenty‐one‐day‐old wild‐type (cultivar Tianlong 1) and transgenic Rhg1‐GmAAT‐OX (Rhg1‐GmAAT‐overexpressing) line gm‐3 were inoculated in quarter‐strength Murashige and Skoog medium containing 50 mm aspartic acid (Asp) and glutamic acid (Glu), 75 mm glutamine (Gln), and 25 mm glycine (Gly). As controls, the wild‐type (cultivar Tianlong 1) plants were grown on quarter‐strength Murashige and Skoog medium only. Images were taken at 3 days after treatment. Scale bar, 5 cm. WT, wild‐type; OX, Rhg1‐GmAAT‐OX line gm‐3. [file MPP-20-270-s005.docx]

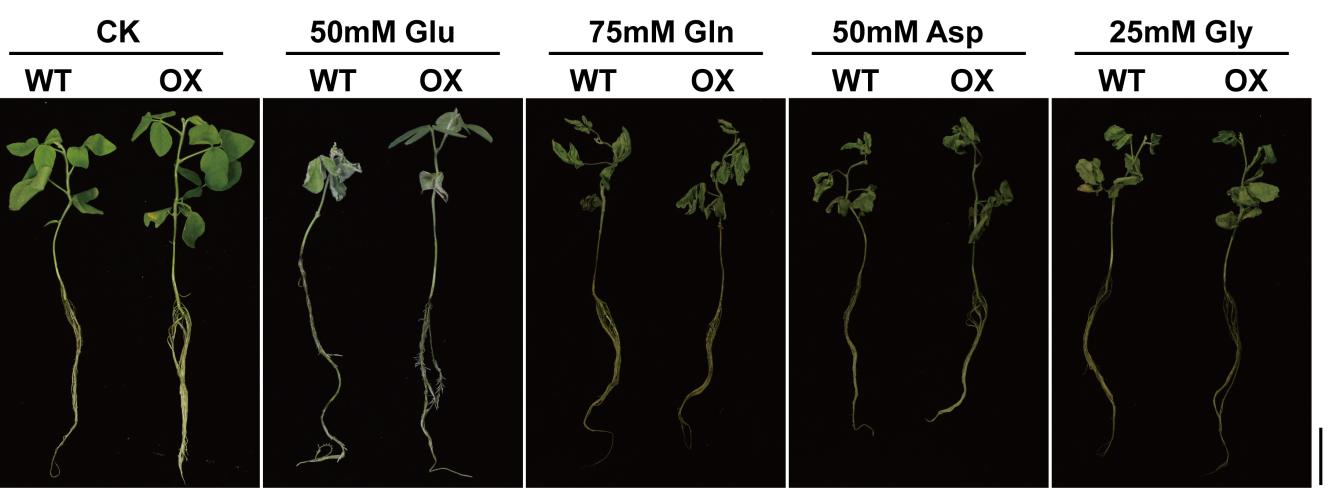


**Figure S5. Growth of soybean seedlings subjected to excess amounts of amino acids.** Twenty-one-day-old wild-type (cultivar Tianlong 1) and transgenic overexpressed Rhg1-GmAAT-OX line gm-3 were inoculated in 1/4 Murashige and Skoog (MS) medium that contained 50 mM aspartate (Asp) and glutamate (Glu), 75 mM glutamine (Gln) and 25 mM glycine (Gly). As controls, the wild-type (cultivar Tianlong 1) plants were grown on 1/4 MS medium only. Images were taken at 3 days after treatment. Scale bar=5 cm. WT, wild-type; OX, Rhg1-GmAAT-OX line gm-3.
