# Supplementary material for: The soybean Rhg1 amino acid transporter gene alters glutamate homeostasis and jasmonic acid‐induced resistance to soybean cyst nematode
Source: Mol Plant Pathol. 2018 Nov 15;20(2):270–86. doi: 10.1111/mpp.12753 (PMC6637870; doi:10.1111/mpp.12753)
Supplement: Supplementary file 6 — Fig. S 6 Responses of a pair of near‐isogenic lines (NILs) to soybean cyst nematodes (SCNs). NIL‐S, NIL‐R and Hutcheson soybean seedlings were transplanted into sterilized sand, after which each plant was inoculated with 2000 J2 nematodes. Female cysts were quantified after 30 days. The experiments were repeated at least three times, each producing similar results. The values are the means ± standard deviations (SDs) (n = 6). Asterisks indicate a statistically significant difference of NIL‐R compared with Hutcheson. **P < 0.01 (multiple t‐test followed by the Holm–Sidak post hoc test). [file MPP-20-270-s006.docx]

**
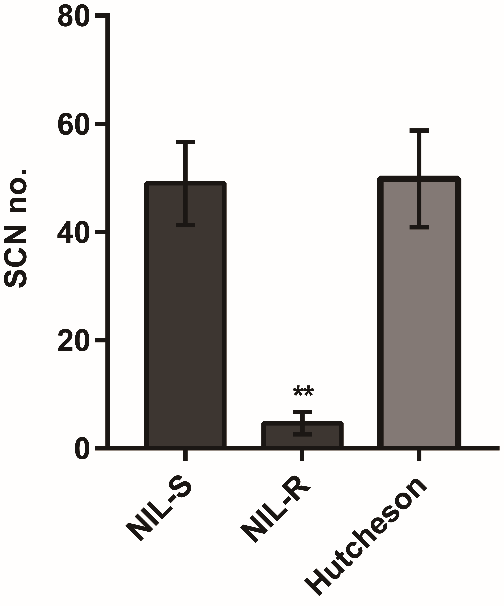
**

**Figure S6 Responses of a pair of near isogenic lines (NILs) to soybean cyst nematodes (SCNs)**. NIL-S, NIL-R and Hutcheson soybean seedlings were transplanted into sterilized sand, after which each plant was inoculated with 2000 J2 nematodes. Female cysts were quantified after 30 days. The experiments were repeated at least three times, each produced similar results. The values are the means±SDs (n=6). Asterisks indicate a statistically significant difference of NIL-R compared with Hutchson. **, P<0.01 (multiple t-test followed by the Holm-Sidak post hoc test)
