# Supplementary material for: The soybean Rhg1 amino acid transporter gene alters glutamate homeostasis and jasmonic acid‐induced resistance to soybean cyst nematode
Source: Mol Plant Pathol. 2018 Nov 15;20(2):270–86. doi: 10.1111/mpp.12753 (PMC6637870; doi:10.1111/mpp.12753)
Supplement: Supplementary file 7 — Fig. S 7 Kyoto Encyclopedia of Genes and Genomes (KEGG) pathway enrichment scatter diagram of up‐regulated differentially expressed genes (DEGs). Only the 20 most enriched pathways are displayed in the diagram. The degree of KEGG pathway enrichment is represented by the rich factor, Q‐value and the number of unigenes enriched in a particular KEGG pathway. The rich factor is the ratio of differentially expressed unigenes enriched in a pathway to the total number of annotated unigenes in that pathway. The greater the rich factor, the greater the degree of enrichment. The Q‐value indicates the corrected P value and ranges from zero to unity; a Q‐value closer to zero indicates more enrichment. [file MPP-20-270-s007.docx]

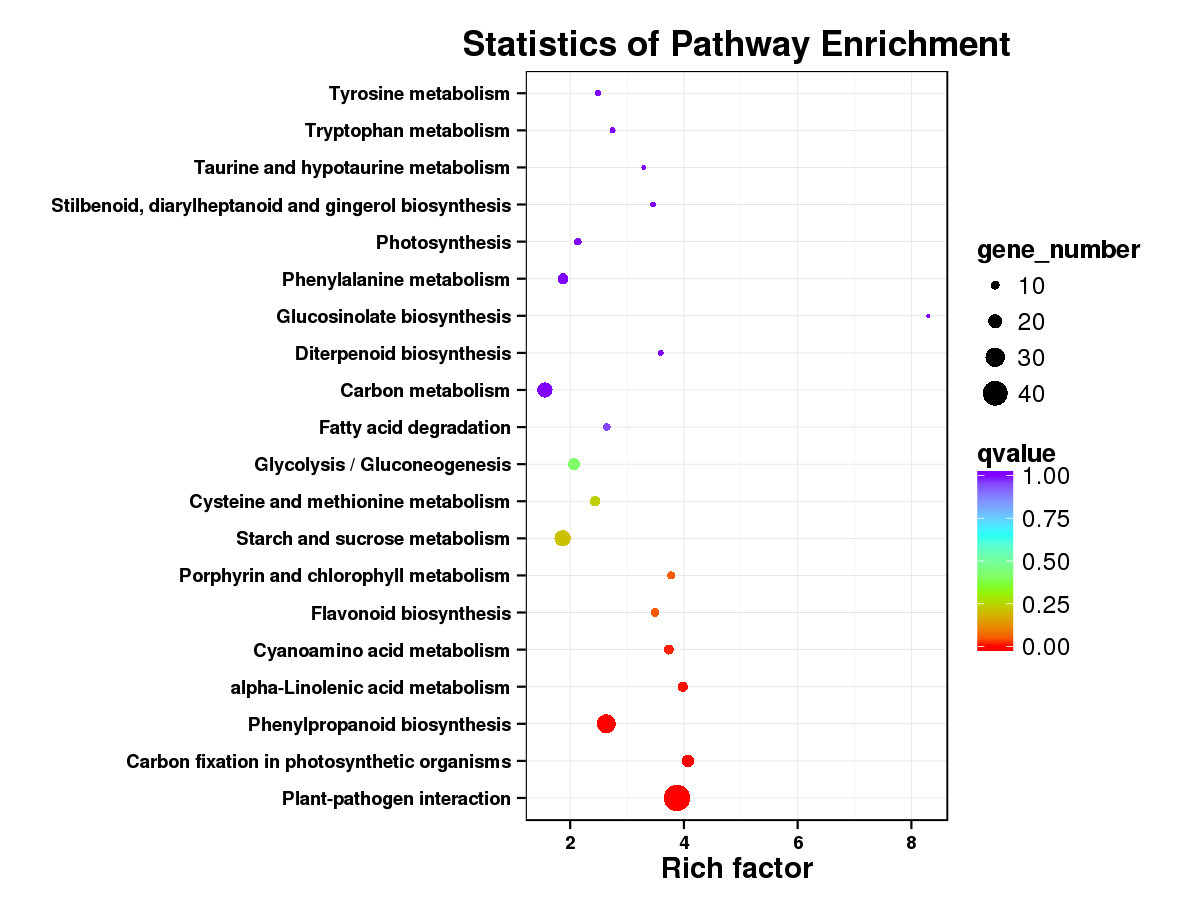


**Figure S7 Kyoto Encyclopedia of Genes and Genomes (KEGG) pathway enrichment scatter diagram of up-regulated differentially expressed genes (DEGs).** Only the 20 most enriched pathways are displayed in the diagram. The degree of KEGG pathway enrichment is represented by rich factor, Q-value, and the number of unigenes enriched in a particular KEGG pathway. The rich factor means the ratio of differentially expressed unigenes enriched in a pathway to the total number of annotated unigenes in that pathway. The greater of the rich factor is, the greater the degree of enrichment. The Q-value indicates the corrected P-value and ranges from 0 and 1, and a Q-value closer to 0 indicates more enrichment.
