# Supplementary material for: The soybean Rhg1 amino acid transporter gene alters glutamate homeostasis and jasmonic acid‐induced resistance to soybean cyst nematode
Source: Mol Plant Pathol. 2018 Nov 15;20(2):270–86. doi: 10.1111/mpp.12753 (PMC6637870; doi:10.1111/mpp.12753)
Supplement: Supplementary file 8 — Fig. S8 Jasmonic acid (JA) content in soybean roots. (a) JA content in a bulk (Bulk‐S) of susceptible soybean varieties (Hutcheson, Magellan and Williams 82) and a bulk (Bulk‐R) of naturally resistant soybean varieties (PI437655, PI495017C, PI209332, PI438503A and PI467312). (b) JA content in the roots of susceptible soybean variety Hutcheson and resistant soybean variety PI88788. Seedlings were cultured in quarter‐strength Murashige and Skoog medium for 4 weeks, after which roots were harvested. Endogenous JA was quantified by following metabolic profiling procedures. The values are the means ± standard deviations (SDs) (n = 6). Asterisks indicate a statistically significant difference of resistant soybean varieties compared with susceptible soybean varieties. **P < 0.01 (multiple t‐test followed by the Holm–Sidak post hoc test). CPS, counts per second. [file MPP-20-270-s008.docx]

**
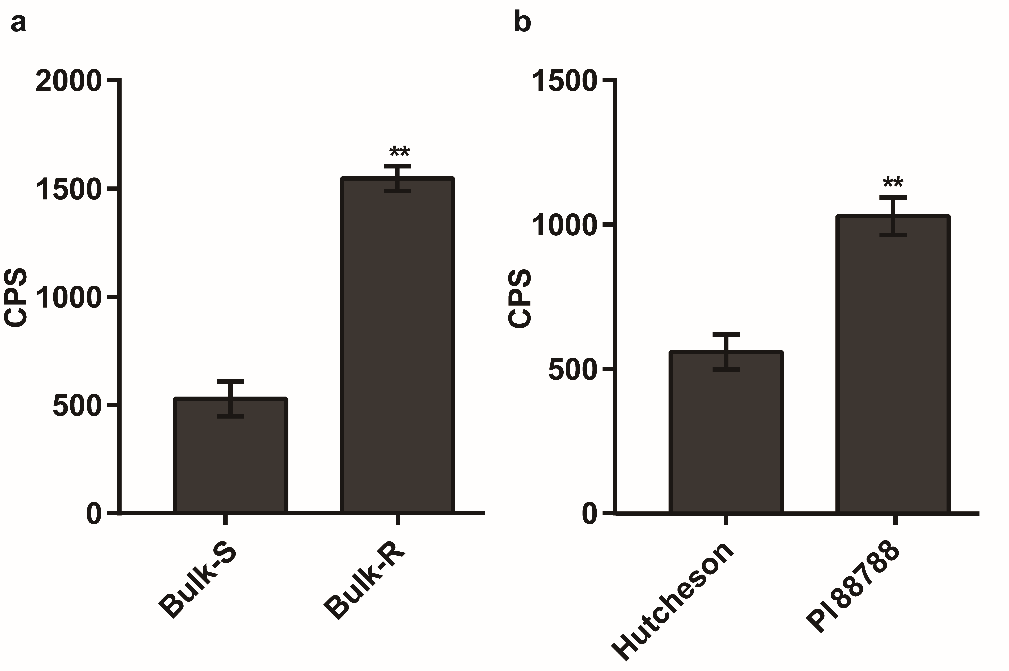
**

**Figure S8. Jasmonic acid (JA) content in the soybean roots.** (a) JA content in a bulk (Bulk-S) of susceptible soybean varieties (Hutcheson, Magellan and Williams 82) and a bulk (Bulk-R) of naturally resistant soybean varieties (PI437655; PI495017C; PI209332; PI438503A; PI467312). (b) JA content in the roots of susceptible soybean variety Hutcheson and resistant soybean variety PI88788. Seedlings were cultured in 1/4 Murashige and Skoog (MS) for 4 weeks, after which roots were harvested. Endogenous JA were quantified by following metabolic profiling procedures. The values were the means±SDs (n=6). Asterisks indicate a statistically significant difference of resistant soybean varieties compared with susceptible soybean varieties. **, P<0.01 (multiple t-test followed by the Holm-Sidak post hoc test). CPS, counts per second
