# Supplementary material for: The soybean Rhg1 amino acid transporter gene alters glutamate homeostasis and jasmonic acid‐induced resistance to soybean cyst nematode
Source: Mol Plant Pathol. 2018 Nov 15;20(2):270–86. doi: 10.1111/mpp.12753 (PMC6637870; doi:10.1111/mpp.12753)
Supplement: Supplementary file 9 — Fig. S9 Expression of jasmonic acid (JA) biosynthesis genes expressed in n‐propyl gallate (nPG)‐treated PI88788 roots. Twelve‐day‐old PI88788 seedlings were inoculated with either water containing 0.02% ethanol (mock) or a 100 μm nPG solution for 3 days. The total RNA was extracted from the roots. The expression levels of the genes of interest were assayed by quantitative reverse transcription‐polymerase chain reaction (qRT‐PCR). The expression levels of all samples were normalized to SKIP16. The values are the means ± standard deviations (SDs) (n = 3). Asterisks indicate a statistically significant difference of nPG‐treated roots compared with mock‐treated roots. *0.01 < P < 0.05, **P < 0.01 (multiple t‐test followed by the Holm–Sidak post hoc test). [file MPP-20-270-s009.docx]

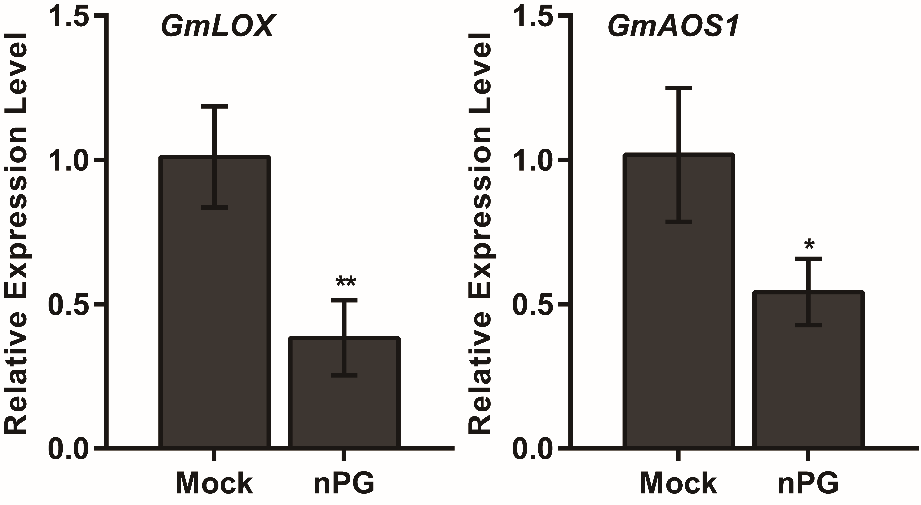


**Figure S9. Expression of JA biosynthesis genes expressed in n-propyl gallate (nPG)-treated PI88788 roots.** Twelve-day--old PI88788 seedlings were inoculated with either water containing 0.02% ethanol (Mock) or a 100 μM nPG solution for 3 days. The total RNA was extracted from the roots. The expression levels of the genes of interest were assayed by quantitative RT-PCR. The expression levels of all samples were normalized to *SKIP16*. The values were the means±SDs (n=3). Asterisks indicate a statistically significant difference of nPG-treated roots compared with Mock-treated roots. *, 0.01<P<0.05; **, P<0.01 (multiple t-test followed by the Holm-Sidak post hoc test).
