# Supplementary material for: The soybean Rhg1 amino acid transporter gene alters glutamate homeostasis and jasmonic acid‐induced resistance to soybean cyst nematode
Source: Mol Plant Pathol. 2018 Nov 15;20(2):270–86. doi: 10.1111/mpp.12753 (PMC6637870; doi:10.1111/mpp.12753)
Supplement: Supplementary file 10 — Fig. S1 0 Expression of Rhg1‐GmWI12 (Glyma.18G022700) induced by jasmonic acid (JA). Twelve‐day‐old Williams 82 soybean seedlings were cultured in quarter‐strength Murashige and Skoog medium that contained either 50 μm JA or water containing 0.02% ethanol (as a control). The roots were sampled after 8 h, after which their total RNA was extracted. The expression of the genes of interest was assayed by real‐time quantitative reverse transcription‐polymerase chain reaction (qRT‐PCR). The expression levels of all samples were normalized to SKIP16. The values are the means ± standard deviations (SDs) (n = 3). Asterisks indicate a statistically significant difference of JA‐treated roots compared with control roots. *0.01 < P < 0.05, **P < 0.01 (multiple t‐test followed by the Holm–Sidak post hoc test). [file MPP-20-270-s010.docx]

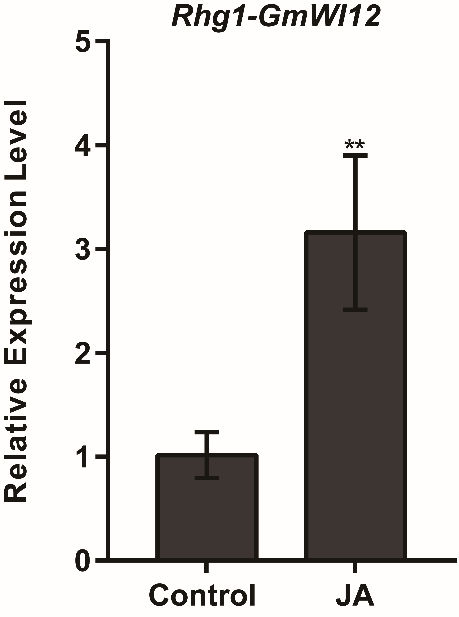


**Figure S10. Expression of *Rhg1-GmWI12* (*Glyma.18G022700*) induced by jasmonic acid (JA).** Twelve-day-old Williams 82 soybean seedlings were cultured in 1/4 Murashige and Skoog (MS) medium that contained either 50 μM JA or water containing 0.02% ethanol (as a control). The roots were sampled after 8 hours, after which their total RNA was extracted. The expression of the genes of interest was assayed by quantitative quantitative real-time PCR. The expression levels of all samples were normalized to *SKIP16*. The values were the means±SDs (n=3). Asterisks indicate a statistically significant difference of the JA-treated roots compared with the control roots. *, 0.01<P<0.05; **, P<0.01 (multiple t-test followed by the Holm-Sidak post hoc test).
