# Supplementary material for: The soybean Rhg1 amino acid transporter gene alters glutamate homeostasis and jasmonic acid‐induced resistance to soybean cyst nematode
Source: Mol Plant Pathol. 2018 Nov 15;20(2):270–86. doi: 10.1111/mpp.12753 (PMC6637870; doi:10.1111/mpp.12753)
Supplement: Supplementary file 11 — Table S1 Free amino acid contents in the roots and leaves of 21‐day‐old wild‐type (cultivar Tianlong 1) and Rhg1‐GmAAT‐overexpressing (Rhg1‐GmAAT‐OX) line gm‐3. Amino acid contents were expressed as nanomoles per milligram. Root and leaf measurements were calculated using the dry weight (DW). Four plants constituted a sample, and three samples per line were used for the experiments. The values are the means ± standard deviations (SDs) (n = 3). The values shown in bold are significantly (P < 0.05) higher than those of the wild‐type (multiple t‐test followed by the Holm–Sidak post hoc test). This table contains the data presented in Fig. 4c,d, as well as additional information about the amino acids not presented in Fig. 4c,d. WT, wild‐type (cultivar Tianlong 1); OX, Rhg1‐GmAAT‐OX line gm‐3. [file MPP-20-270-s011.docx]

**Table S1. Free amino acid contents in the roots and leaves of 21-day-old wild-type (cultivar Tianlong 1) and Rhg1-GmAAT-OX line gm-3.** Amino acid contents were expressed as nanomoles per milligram. Root and leaf measurements were calculated using the dry weight (DW). Every 4 plants constituted a sample, and three samples per line were used for the experiments. The values were the means±SDs (n=3). The values shown in bold are significantly (p<0.05) higher than those of wild-type (multiple t-test followed by the Holm-Sidak post hoc test). This table contains the data presented in Figure 4c and 4d as well as additional information about the amino acids not presented in Figure 4c and 4d. WT, wild-type (cultivar Tianlong 1); OX, Rhg1-GmAAT-OX line gm-3.

| **Amino acid** | **Roots** | |  | **Leaves** | |
| --- | --- | --- | --- | --- | --- |
|  | **WT** | **OX** |  | **WT** | **OX** |
| **Ser** | 6.907±0.369 | **5.427±0.141** |  | 13.389±1.028 | 13.291±2.490 |
| **Gly** | 2.339±0.165 | **1.606±0.07** |  | 1.728±0.215 | 1.531±0.351 |
| **His** | 2.958±0.375 | 2.341±0.216 |  | 8.266±1.313 | 10.529±1.793 |
| **Thr** | 3.747±0.159 | **2.716±0.126** |  | 3.346±0.116 | 2.779±0.556 |
| **Glu** | 2.645±0.217 | **7.619±0.284** |  | 0.948±0.152 | **0.572±0.014** |
| **Gln** | 19.962±1.167 | **13.264±0.137** |  | 11.756±0.666 | 10.850±1.965 |
| **Asp** | 19.098±0.744 | 18.998±0.253 |  | 22.709±0.803 | 18.822±3.808 |
| **Asn** | 192.576±10.075 | 202.579±4.811 |  | 88.981±1.805 | 89.861±16.49 |
| **Ala** | 10.581±0.073 | **7.440±0.208** |  | 15.787±0.823 | 13.767±2.696 |
| **Arg** | 8.971±1.178 | 8.224±0.612 |  | 28.583±2.882 | 20.362±5.141 |
| **Pro** | 1.411±0.079 | **1.059±0.053** |  | 2.939±0.237 | 2.361±0.489 |
| **Cys** | 0.0179±0.017 | 0.023±0.041 |  | 0.045±0.006 | 0.034±0.006 |
| **Lys** | 5.0210±1.014 | 4.278±0.147 |  | 5.849±0.651 | 3.857±0.901 |
| **Met** | 0.410±0.017 | **0.101±0.002** |  | 0.965±0.074 | 0.881±0.157 |
| **Val** | 3.043±0.059 | **2.145±0.099** |  | 3.322±0.248 | 2.852±0.474 |
| **Tyr** | 1.0710±0.027 | **0.564±0.012** |  | 1.547±0.133 | 1.410±0.364 |
| **Ile** | 1.198±0.040 | **0.682±0.032** |  | 1.494±0.161 | 1.461±0.318 |
| **Leu** | 2.370±0.055 | **1.345±0.044** |  | 3.691±0.396 | 3.079±0.571 |
| **Phe** | 2.321±0.129 | **1.034±0.013** |  | 2.608±0.291 | 3.442±0.864 |
| **Trp** | 0.440±0.032 | **0.228±0.011** |  | 0.490±0.045 | 0.750±0.205 |
| **TOTAL** | 287.087±12.089 | 281.672±5.794 |  | 218.443±9.682 | 203.325±38.534 |
