# Supplementary material for: The soybean Rhg1 amino acid transporter gene alters glutamate homeostasis and jasmonic acid‐induced resistance to soybean cyst nematode
Source: Mol Plant Pathol. 2018 Nov 15;20(2):270–86. doi: 10.1111/mpp.12753 (PMC6637870; doi:10.1111/mpp.12753)
Supplement: Supplementary file 12 — Table S2 RNA‐sequencing (RNA‐Seq) results regarding changes in the expression of jasmonic acid (JA)‐related genes and Rhg1 genes on Rhg1‐GmAAT overexpression. The values are the means ± standard deviations (SDs) (n = 3). FDR, false discovery rate. [file MPP-20-270-s012.docx]

**Table S2. RNA-Seq results regarding changes in the expression of jasmonic acid (JA)-related genes and *Rhg1* genes upon *Rhg1-GmAAT* overexpression.** The values were the means±SDs (n=3)**.** FDR, False discovery rate.

| **Gene** | **Locus ID** | **Fold change (log2 ratio)** | **FDR** |
| --- | --- | --- | --- |
| **Jasmonic acid biosynthesis** |  |  |  |
| *GmLOX* | *Glyma.03G264300* | 1.16 | 1.88E-11 |
|  | *Glyma.19G263300* | 1.13 | 1.69E-09 |
|  | *Glyma.07G039900* | 0.93 | 7.12E-10 |
|  | *Glyma.16G008700* | 0.52 | 1.29E-03 |
| *GmAOS1* | *Glyma.14G078600* | 2.29 | 3.96E-28 |
| *GmAOS2* | *Glyma.17G246500* | 1.94 | 2.05E-14 |
| *GmOPR3* | *Glyma.13G109700* | 0.90 | 1.05E-12 |
|  | *Glyma.17G049900* | 3.28 | 1.75E-55 |
| **Jasmonic acid**  **signaling pathway** |  |  |  |
| *VSPβ* | *Glyma.08G200100* | -1.78 | 1.04E-04 |
| *GmJAZ1* | *Glyma.11G038600* | 3.24 | 1.13E-32 |
|  | *Glyma.01G204400* | 2.70 | 1.89E-48 |
|  | *Glyma.17G047700* | 2.91 | 1.61E-21 |
| *GmbHLH35* | *Glyma.17G058600* | 1.76 | 9.11E-21 |
| *GmMPK3* | *Glyma.12G073000* | 1.47 | 1.73E-17 |
|  | *Glyma.U021800* | 0.92 | 0.000233 |
| ***Rhg1* gene** |  |  |  |
| *GmSNAP18* | *Glyma.18G022500* | 0.15 | 0.47 |
| *Rhg1-GmWI12* | *Glyma.18G022700* | 0.95 | 1.16E-09 |
