# Supplementary material for: The soybean Rhg1 amino acid transporter gene alters glutamate homeostasis and jasmonic acid‐induced resistance to soybean cyst nematode
Source: Mol Plant Pathol. 2018 Nov 15;20(2):270–86. doi: 10.1111/mpp.12753 (PMC6637870; doi:10.1111/mpp.12753)
Supplement: Supplementary file 13 — Table S3 Metabolite analysis results regarding changes in hormone contents on Rhg1‐GmAAT overexpression. The values are the means ± standard deviations (SDs) (n = 3). CPS, counts per second; VIP, variable importance in projection. [file MPP-20-270-s013.docx]

**Table S3. Metabolite analysis results regarding changes in hormone contents upon *Rhg1-GmAAT* overexpression.** The values were the means±SDs (n=3). CPS, counts per second; VIP, Variable importance in projection.

| **Compounds** | **Class** | **Response value (CPS)** | | **Fold Change (log2 ratio)** | **VIP** |
| --- | --- | --- | --- | --- | --- |
|  |  | **Tianlong1** | **gmOX-3**  **(*Rhg1-GmAAT*)** |  |  |
| (+)-Jasmonic acid (JA) | Phytohormones | 3773.33±285.7 | 9806.67±3027.59 | 2.60 | 2.01 |
| tZOG | Phytohormones | 122033.33±25620.35 | 55866.67±15834.42 | 0.46 | 1.73 |
| Methoxy indoleacetic acid | Phytohormones | 909000±195807.73 | 437666.67±238479.91 | 0.48 | 1.66 |
| 5-Methoxy-3-indoleacetate | Phytohormones | 861000±205768.48 | 392333.33±243050.52 | 0.46 | 1.71 |
