# Supplementary material for: The soybean Rhg1 amino acid transporter gene alters glutamate homeostasis and jasmonic acid‐induced resistance to soybean cyst nematode
Source: Mol Plant Pathol. 2018 Nov 15;20(2):270–86. doi: 10.1111/mpp.12753 (PMC6637870; doi:10.1111/mpp.12753)
Supplement: Supplementary file 14 — Table S4 Oligonucleotide primers used in this study. [file MPP-20-270-s014.docx]

**Table S4. Oligonucleotide primers used in this study.**

| **Name** | **Sequence (5’-3’)** |
| --- | --- |
| **Expression Vectors** | |
| *GmAAT-Fw* | ATGTCTCCGGCCGCCGGAGTCAGC |
| *GmAAT-Rw* | TTATGACTTGCTACTAAAAGCATTATAT |
| *PromGmAAT-Fw* | GACACTAAATCAGCATTTTGAGGAGACAC |
| *PromGmAAT-Rw* | GCCGGAGGCAAAATTGTGAGGATCGGAG |
| *GmAAT-GFP(AgeI)-Fw* | tctagcgctaccggt ATGTCTCCGGCCGCCGGAG |
| *GmAAT-GFP(AgeI)-Rw* | atggtggcgaccggtag TGACTTGCTACTAAAAGCATTATATATGTTG |
| *GmAAT-GFP(BamHI)-Fw* | ggtaccgcgggcccgggatcc ATGTCTCCGGCCGCCGGAG |
| *GmAAT-GFP(BamHI)-Rw* | cggcagcggcagcagccggatccTGACTTGCTACTAAAAGCATTATATATGTTG |
| **Transcript Abundance Using RT-PCR or qRT-PCR** | |
| *GmAAT-RT-F* | CTGGTTCAGCAGTTGGTTCCTTG |
| *GmAAT-RT-R* | GTCTCTTCTCGTTGATATACCTTTAAC |
| *ATACT7-RT-F* | GGTGAGGATATTCAGCCACTTGTCTG |
| *ATACT7-RT-R* | TGTGAGATCCCGACCCGCAAGATC |
| *GmAAT-qRT-F* | CGTGTAGAGTCCTTGAAGTACAGC |
| *GmAAT-qRT-R* | ACCAGAGCTGTGATAGCCAACC |
| *GmLOX1-qRT-F* | CGCTTCAAGCCAATGGGAAT |
| *GmLOX1-qRT-R* | ACACCTGCCAAAGTTTCCCT |
| *GmAOS1-qRT-F* | CCCTCCAGAACAAAGAAACGA |
| *GmAOS1-qRT-R* | CTTGGCTCAGGGGAGGTAAC |
| *GmOPR3-qRT-F* | CCAAGGGCACGCCTTTAACT |
| *GmOPR3-qRT-R* | GCAGCTTGCAGGCAATATAGATG |
| *GmPDF1.2-qRT-F* | CAAGAACATGGGACTCACGC |
| *GmPDF1.2-qRT-R* | CGTGACAGGCTCCATGAGTT |
| *GmJAZ1-qRT-F* | GCTGCCAGAGCACCATATC |
| *GmJAZ1-qRT-R* | GCCATTAGCAACAGCTAAA |
| *GmbHLH-qRT-F* | AGTCACATCCCTCATTGCCG |
| *GmbHLH-qRT-R* | CCACATGAGGGCAACTTGGA |
| *GmSKIP16-qRT-F* | GAGCCCAAGACATTGCGAGAG |
| *GmSKIP16-qRT-R* | CGGAAGCGGAAGAACTGAACC |
| *ATACT2-qRT-F* | AGTGTCTGGATCGGTGGTTC |
| *ATACT2-qRT-R* | CCCCAGCTTTTTAAGCCTTT |
| *Glyma.06G233600-qRT-F* | TGACGTATCTCGAGCAACCC |
| *Glyma.06G233600-qRT-R* | AAGGTTGAGGGCCTAGTTGC |
| *Glyma.07G104500-qRT-F* | CTGACTCACTTATAAAAGGCGC |
| *Glyma.07G104500-qRT-R* | CAATAGGTGCAAGCTCTAGGTA |
| *AtGLR2.7-qRT-F* | CGAGACTTGCGATTCACATTAG |
| *AtGLR2.7-qRT-R* | CACTTGCTCGTTCTTGATTAGG |
| *AtGSR1-qRT-F* | GAGACACAAAGAACACATTGCT |
| *AtGSR1-qRT-R* | AAAGGAAAGTGTTGATGTCAGC |
