# Supplementary material for: The soybean Rhg1 amino acid transporter gene alters glutamate homeostasis and jasmonic acid‐induced resistance to soybean cyst nematode
Source: Mol Plant Pathol. 2018 Nov 15;20(2):270–86. doi: 10.1111/mpp.12753 (PMC6637870; doi:10.1111/mpp.12753)
Supplement: Supplementary file 15 — Methods S1 Multiple alignments. [file MPP-20-270-s015.docx]

**Methods S1**

***Multiple alignments***

The sequence of the GmAAT protein was queried against all the annotated proteins of *Arabidopsis thaliana* (https://www.arabidopsis.org/index.jsp) using BLASTP (version 2.2.26) with an E-value setting of 1e-5.
