# Supplementary material for: The soybean Rhg1 amino acid transporter gene alters glutamate homeostasis and jasmonic acid‐induced resistance to soybean cyst nematode
Source: Mol Plant Pathol. 2018 Nov 15;20(2):270–86. doi: 10.1111/mpp.12753 (PMC6637870; doi:10.1111/mpp.12753)
Supplement: Supplementary file 16 — Methods S2 DNA construct. [file MPP-20-270-s016.docx]

**Methods S2**

***DNA construct***

The *GmAAT* cDNA was cloned into a pEZS-NL vector (Song et al., 2012) in the *BamH I* restriction site by using a ClonExpress II One Step Cloning Kit (Vazyme, China) to generate P_35S_-GmAAT-GFP. The final vector was transiently expressed in onion epidermal cells by using particle bombardment technology as described by Taylor et al. (2008). The construct P_35S_-GmAAT-GFP using the pEGAD-GFP vector was expressed in soybean hairy roots. For nuclei staining, DAPI reagent (4, 6-diamidino-2-phenylindole) (Sigma, USA) was used. The onion epidermises or tobacco epidermises were incubated in 5μg/ml of DAPI for 5 min. GFP and DAPI were observed using a Nikon A1 spectral confocal microscope (Nikon, Japan) at an excitation wavelength of 488 nm and an emission wavelength of 509 nm for GFP, and at an excitation wavelength of 405 nm and an emission wavelength of 492 nm for DAPI.
