# Supplementary material for: The soybean Rhg1 amino acid transporter gene alters glutamate homeostasis and jasmonic acid‐induced resistance to soybean cyst nematode
Source: Mol Plant Pathol. 2018 Nov 15;20(2):270–86. doi: 10.1111/mpp.12753 (PMC6637870; doi:10.1111/mpp.12753)
Supplement: Supplementary file 17 — Methods S3 RNA‐sequencing (RNA‐Seq): sample preparation and data analysis. [file MPP-20-270-s017.docx]

**Methods S3**

***RNA-Seq: sample preparation and data analysis***

The total RNA was extracted from the roots of wild-type (cultivar Tianlong 1) or Rhg1-GmAAT-OX plants using Trizol reagent (Invitrogen, USA). Three biological replicates per line were included. In accordance with the manufacturer’s instructions, an equal amount of total RNA from each sample, which consisted of 10 soybean roots, was pooled for RNA-Seq to obtain a comprehensive range of transcripts (mRNA sequencing, sample preparation guide; Illumina, USA). The sample library was qualified and quantified using an Agilent 2100 Bioanalyzer and an ABI Step OnePlus Quantitative real-time PCR System, after which the library was subjected to sequencing by an Illumina HiSeq 2000 (Illumina, USA). The raw data were cleaned to high-quality (clean) reads by filtering adaptor reads and low-quality reads (bases having a quality value≤5). The clean reads were mapped to the latest version of the soybean reference genome (*Glycine max* Wm82.a2.v1) (Goodstein et al., 2012) downloaded from Phytozome using SOAP aligner/SOAP2 (Li et al., 2009). Significant differentially expressed genes (DEGs) were identified using a false discovery rate (FDR)≤0.01 and an absolute value of log2 ratio≥1.
