# Supplementary material for: The soybean Rhg1 amino acid transporter gene alters glutamate homeostasis and jasmonic acid‐induced resistance to soybean cyst nematode
Source: Mol Plant Pathol. 2018 Nov 15;20(2):270–86. doi: 10.1111/mpp.12753 (PMC6637870; doi:10.1111/mpp.12753)
Supplement: Supplementary file 18 — Methods S4 Metabolic profiling. [file MPP-20-270-s018.docx]

**Methods S4**

***Metabolic profiling***

The roots of the wild-type (cultivar Tianlong 1) and Rhg1-GmAAT-OX plants were cultured in 1/4 Murashige and Skoog (MS) medium. After 4 weeks, the roots were harvested, rinsed briefly in phosphate buffered solution (PBS), and immediately frozen in liquid nitrogen. Each sample, which consisted of 10 soybean roots, was freeze-dried and extracted as previously described prior to being analyzed by a liquid chromatography-electrospray ionization coupled to tandem mass spectrometry (LC-ESI-MS/MS) system (Chen et al., 2013). A previously reported relative quantification method was used to analyze the samples (Chen et al., 2014). Three biological replicates per line were analyzed. The Principal Component Analysis (PCA) and Partial Least Squares-Discriminant Analysis (PLS-DA) were used to analyze the collected data to identify variant metabolites. From the multivariate analysis of the Variable Importance in Projection (VIP) parameters based on the PLS-DA model. High value of VIP score indicates great contribution of the proteins to the group separation. A VIP score of 1 is considered to enable discrimination between 2 phenotypes. The metabolites of different varieties were preliminarily screened out (VIP≥1). The differential metabolites were further identified by using fold change (fold change≥2 and fold change≤0.5). A p value was also used to measure the significant difference between samples. Metabolic profiling was conducted by Metware company (China).
